# Imbalanced Oxidative Stress Causes Chlamydial Persistence during Non-Productive Human Herpes Virus Co-Infection

**DOI:** 10.1371/journal.pone.0047427

**Published:** 2012-10-15

**Authors:** Bhupesh K. Prusty, Linda Böhme, Birgit Bergmann, Christine Siegl, Eva Krause, Adrian Mehlitz, Thomas Rudel

**Affiliations:** 1 Biocenter, Chair of Microbiology, University of Würzburg, Würzburg, Germany; 2 Heinrich Pette Institute, Leibniz Institute for Experimental Virology, Hamburg, Germany; University of California Merced, United States of America

## Abstract

Both human herpes viruses and *Chlamydia* are highly prevalent in the human population and are detected together in different human disorders. Here, we demonstrate that co-infection with human herpes virus 6 (HHV6) interferes with the developmental cycle of *C. trachomatis* and induces persistence. Induction of chlamydial persistence by HHV6 is independent of productive virus infection, but requires the interaction and uptake of the virus by the host cell. On the other hand, viral uptake is strongly promoted under co-infection conditions. Host cell glutathione reductase activity was suppressed by HHV6 causing NADPH accumulation, decreased formation of reduced glutathione and increased oxidative stress. Prevention of oxidative stress restored infectivity of *Chlamydia* after HHV6-induced persistence. We show that co-infection with Herpes simplex virus 1 or human Cytomegalovirus also induces chlamydial persistence by a similar mechanism suggesting that *Chlamydia* -human herpes virus co-infections are evolutionary shaped interactions with a thus far unrecognized broad significance.

## Introduction

The Gram-negative, obligate intracellular bacterium *Chlamydia trachomatis* is the leading infectious cause of blindness and the third most frequent sexually transmitted infection worldwide. *Chlamydia (Chlamydophila) pneumoniae,* a highly prevalent pathogen with up to 80% serum positivity in adults, is the cause of pneumonia in humans, but has also been associated with chronic diseases like atherosclerosis, progressive neurological disorders and lung cancer [1], [2], [3]. *Chlamydia* has a biphasic developmental cycle with infectious but metabolically inert elementary bodies (EB), which differentiate into larger, metabolically active non-infectious reticulate bodies (RB). At the end of the life cycle, RB re-differentiates into infectious EB to start a new round of infection. *Chlamydia* enters into a persistent phase when exposed to adverse physiological conditions (e.g. amino acid starvation, iron deficiency), and if treated with antibiotics or interferon-γ (IFN-γ) [4]. This phase is characterized by bacterial genome replication without bacterial division or production of infectious bacteria, leading to formation of enlarged so called aberrant bodies [5]. Also co-infection with Herpes simplex virus 1 and -2 (HSV-1, -2) induces persistence of *C. trachomatis* [6], [7], [8], [9]. So far, mechanisms underlying virus-mediated persistence have not been defined.

Human herpes virus 6 (HHV6) belongs to the β-herpesvirus family and is a ubiquitous pathogen showing more than 90% serum positivity in healthy adults. It has CD4+ T-lymphocyte specificity but has been shown to infect many different cell types *in vitro* [10]. Usually asymptomatic, HHV6 is associated with the common, self-limited childhood illness roseola infantum, and rarely with more severe syndromes. In immune compromised patients, reactivation of viral activity may lead to severe limbic encephalitis. There are two subtypes of HHV6, HHV6A and HHV6B with similar infection characteristics but different tissue tropism [10]. The virus persists either in a lytic or latent phase inside the host cell.

Epidemiological studies have connected HHVs and *Chlamydia* in several *in vivo* conditions. HSV2 is associated with *C. trachomatis* in endometritis and acute salpingitis [11]. HHV6 has long been one of the most probable candidates for the development of autoimmune disorders like multiple sclerosis (MS). Co-infection of *C. pneumoniae* is observed in MS [12] and in chronic fatigue syndrome (CFS) patients [13].

Here, we studied the survival and infectivity of *C. trachomatis* and HHV6 in a co-infection model. Our data suggests that HHV6 infection modulates cellular glutathione reductase (GSR) activity leading to increased oxidative stress and decreased levels of reduced glutathione (GSH). We show that these conditions induce chlamydial persistence providing the first mechanistic insight into how herpes virus co-infections affect the infectivity of *Chlamydia*.

## Results

### HHV6 Induces Persistence of *Chlamydia trachomatis* in Epithelial Cells

**Figure 1:**
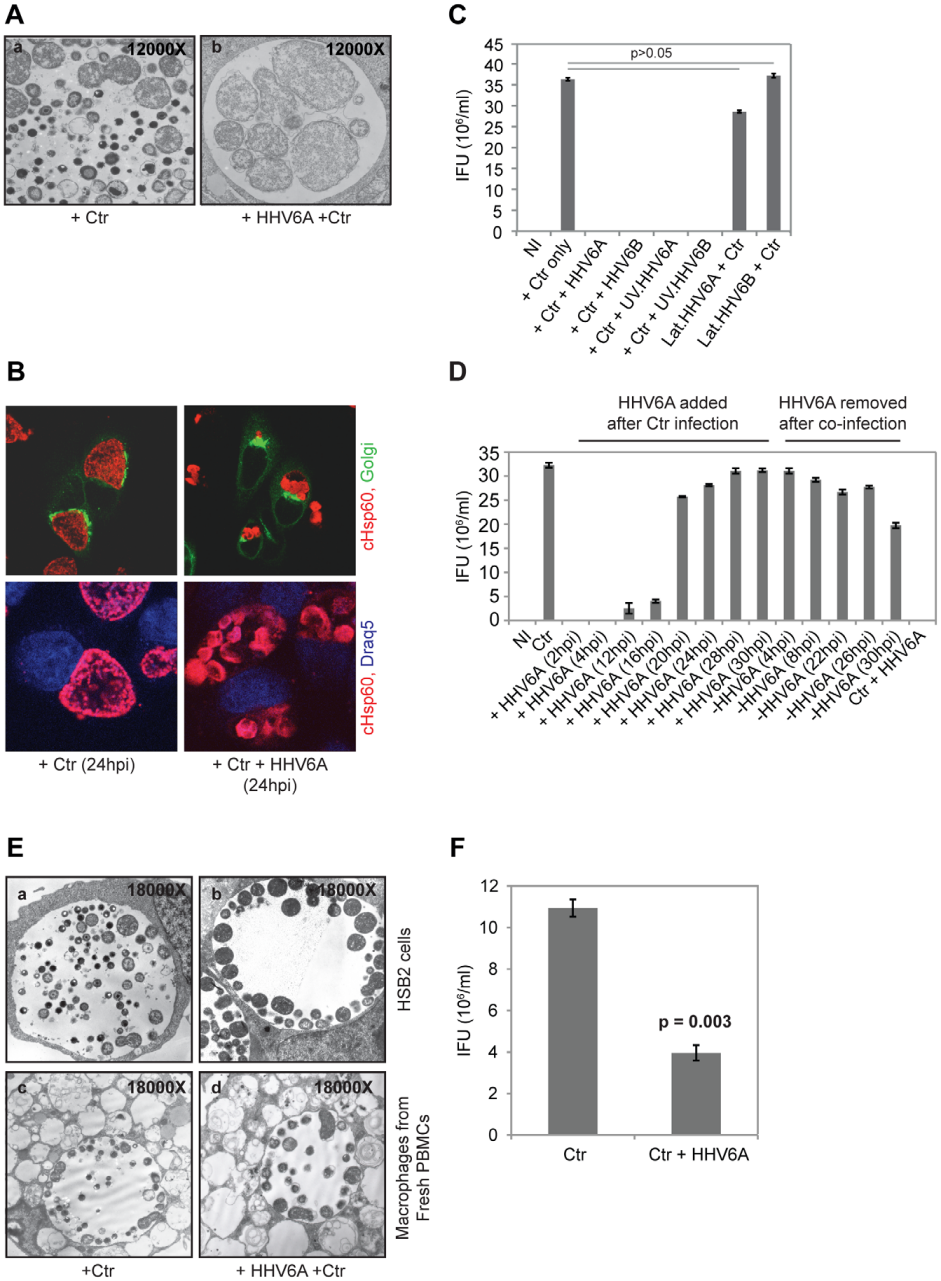
Co-infection of HHV6 induces persistence of *C. trachomatis.* (**A**) Morphology of *Chlamydia*. Transmission electron microscopy (TEM) images of chlamydial inclusions. (a) Single *Chlamydia* infection, (b) co-infection with HHV6A. (**B**) Chlamydial inclusions of co-infected cells differ in morphology compared to single infected cells. Chlamydia was stained with an antibody against cHsp60 (red) and the host cell nuclei were visualized by Draq5 staining (blue). In the upper panel, cells were additionally transfected with Golgi-GFP fusion protein constructs. Samples were viewed under a confocal laser microscopy. (**C**) Co-infection with HHV6A and -6B induces chlamydial persistence. HeLa cells were infected with *C. trachomatis* (Ctr) alone or together with either HHV-6A, -6B, UV inactivated HHV6A (UV.HHV6A) or -HHV6B (UV.HHV6B). In parallel, latent HHV6 (lat.HHV6A and lat.HHV6B) containing HeLa cells were infected with Ctr. The bars indicate inclusion-forming units (IFU) obtained after standard infectivity assays as described in Materials and Methods. Data represent the mean ± SEM of three independent infection experiments. (**D**) Early co-infection with HHV6 is necessary for inducing chlamydial persistence. HeLa cells were infected with *Chlamydia* for 2 h prior to the addition of viral particles for different time points as indicated. In a parallel infection set up, HHV6A was added to *Chlamydia-*infected cells after 2 h, but subsequently HHV6 was removed from the infection media at the indicated time points. Infectivity assays were performed as described in materials and methods. NI: no infection. Data represent the mean ± SEM of three independent infection experiments. (**E**) TEM images of chlamydial inclusions in HSB2 cells (a, b) and in monocyte-derived macrophages from healthy blood donors (c, d). Cells were either infected with Ctr alone (a, c) or together with HHV6A (b, d). (**F**) Chlamydial infectivity is down regulated in monocyte-derived macrophages in presence of HHV6A co-infection. Freshly isolated macrophages were infected with *C. trachomatis* (Ctr) alone or together with HHV6A and chlamydial infectivity was determined. Data represent the mean ± SEM of three independent samples.

To investigate the possible interaction between HHV6 and *Chlamydia* during *in vitro* co-infections, we infected HeLa cells simultaneously with either HHV6 strain U1102 (HHV6A) or Z29 (HHV6B) at 5–10 infectious units per cell and *C. trachomatis* LGV L2 at a multiplicity of infection (MOI) of 1. After 24 h of infection, electron microscopic analysis revealed formation of aberrant inclusions in co-infected cells, which were absent in single *Chlamydia*-infected cells (Figure 1A, B). While normal bacterial inclusions contained EBs and RBs, morphologically altered inclusions of co-infected cells were filled with large so-called aberrant bodies reminiscent of persistent *Chlamydia* (Figure 1A,B). Chlamydial persistence has been shown to coincide with reduced bacterial infectivity [14]. We therefore infected HeLa cells for 48 h and used the lysates to re-infect fresh cells (secondary infection assay or infectivity assay). Secondary infections under standard conditions yielded more than 3.5×10^6^ inclusion forming units in 5×10^5^ cells. In contrast, no inclusions were formed when lysates from co-infected cells were used, indicating a complete loss of infectivity (Figure 1C). Such a dramatic loss of infectivity was not observed in co-infections with other members of the order *Chlamydiales*. HHV6 co-infection with *C. pneumoniae* or *Simkania negevensis* (SN) induced no obvious, aberrant inclusions of primary infected cells (data not shown). But, infectivity was reduced by 36.4% and 59.1% in co-infections with *C. pneumoniae* and HHV6A or 6B, respectively, and 15.0% in co-infections of SN and HHV6A (Figure S1A,B), showing that HHV6’s influence on the *Chlamydiales* infectivity differs during co-infection.

To further characterize the onset of persistence during co-infection, HHV6 was added at different time points to *Chlamydia*-infected cells and infectivity was determined. Loss of infectivity or strongly reduced infectivity was observed if the virus was added up to 16 h after infection with *C. trachomatis*. While addition of the virus at 20 h post infection (p.i.) and 24 h p.i. resulted in 20% and 12% loss of infectivity, respectively, no obvious effect was observed when the virus was added at later time points (Figure 1D, S1C). Interestingly, loss of infectivity caused by co-infection with HHV6 was reversible since removal of the virus from the supernatants as late as 26 to 30 h p.i. restored infectivity (Figure 1D, S1C). These data demonstrated that presence of HHV6 in the early phase of the developmental cycle drives *Chlamydia* into a non-infectious phase.

HHV6 is a lymphotrophic virus [15] and *C. trachomatis* L2 causes lymphogranuloma venerum and has been demonstrated to grow in lymphocytes and macrophages [16], One of the most possible sites for *in vivo* co-infection are therefore human blood cells including macrophages and T-cells. Hence, we first tested the co-infection in T-cell derived HSB2 cells, which allows productive HHV6A infection. We observed similar persistent chlamydial infections in these cells when co-infected with HHV6A (Figure 1E). We then isolated peripheral blood mononuclear cells (PBMCs) from 5 healthy individuals and used them for infection either with *C. trachomatis* alone or together with HHV6A. Monocyte derived macrophages were separated in all these samples and were infected separately (see Material and Methods). We observed mostly persistent chlamydial infection in majority of the infected macrophages (Figure 1E) in presence of HHV6A co-infection, whereas macrophages allowed productive chlamydial infection in the absence of HHV6A (Figure 1E). In freshly isolated PBMCs, chlamydial infection was predominant in macrophages with very few other cell types also showing chlamydial infection. It is noteworthy that the aberrant RBs in persistent chlamydial inclusions were comparatively smaller in size in monocyte-derived macrophages than in cultured epithelial cells. We performed chlamydial infectivity assay in macrophages, which confirmed the visual observations of chlamydial persistence in the presence of HHV6A (Figure 1F). As chlamydial infection is inefficient and asynchronous in suspension cells, we could not perform infectivity assay in the rest of the leukocytes separated from PBMCs. Thus these data supports our hypothesis that human blood cells, especially macrophages and T cells, can allow natural co-infections thus leading to persistent chlamydial life cycle.

### Virus-induced Persistence is Independent of Interferon (IFN) and Allows Long-term Chlamydial Replication

Microarray expression profiling of various IFNs, under different infection conditions was used to rule out host cell anti-viral responses, which may influence chlamydial survival and infectivity. mRNA expression pattern of various IFNs between single *Chlamydia* infections and co-infections remained unaltered (Figure S2A). We could also exclude any soluble factor from the HHV6 producing cell line HSB2/Molt-3, since only cell supernatants harvested before virus release (around 4–5 days post HHV6 infection) did not induce chlamydial persistence (data not shown). Further, supernatants from HSB2/Molt-3 prepared after removing the virus by centrifugation or precipitation failed to induce chlamydial persistence (data not shown). We nevertheless used HHV6 stocks purified by ultra-centrifugation for all further co-infection experiment to avoid any possible contamination from the virus stock.

**Figure 2:**
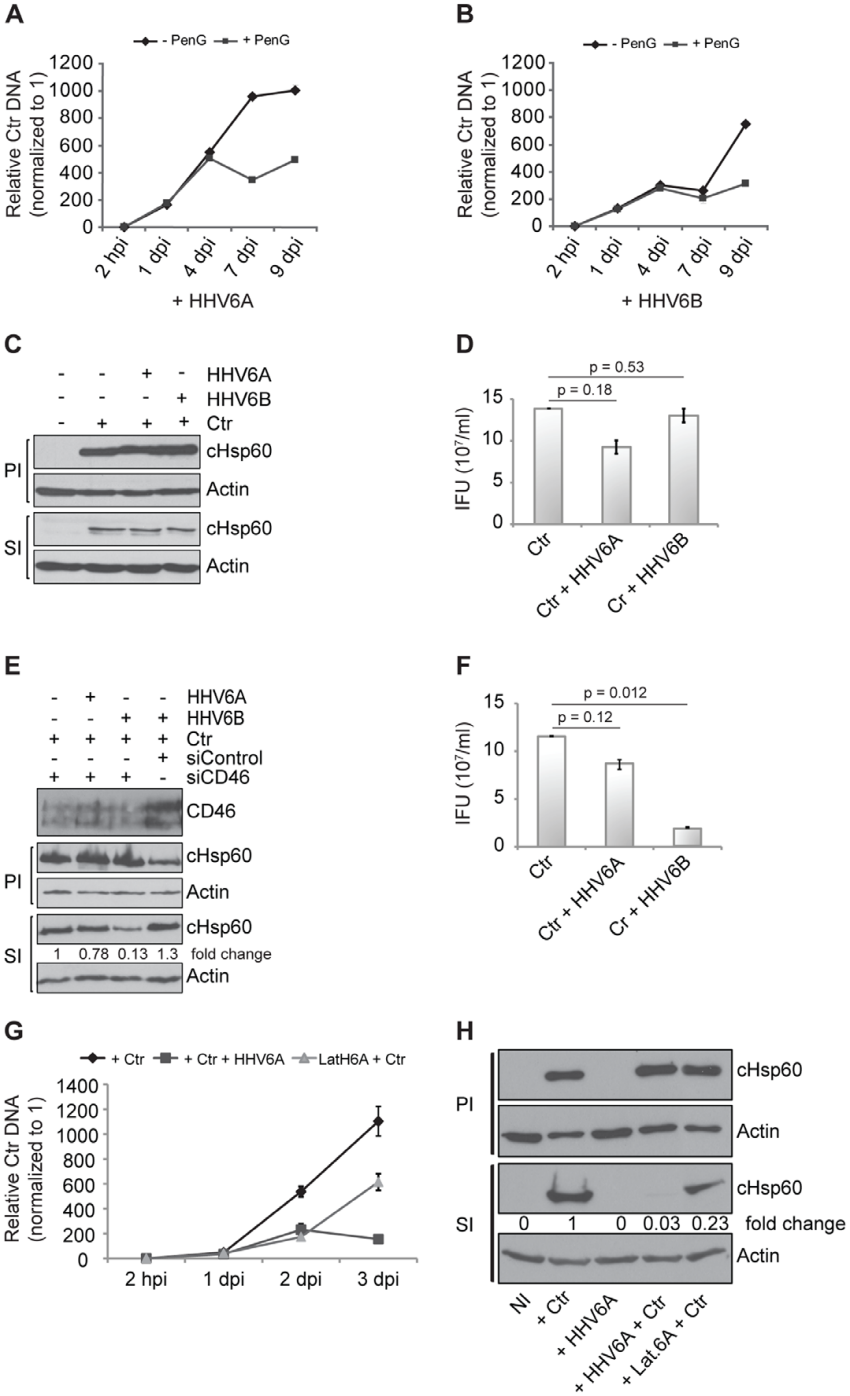
HHV6-mediated chlamydial persistence differs from other forms of chlamydial persistence and depends on viral entry. (**A,B**) HHV6-mediated chlamydial persistence differs from that induced by Penicillin G. HeLa cells were co-infected with *Chlamydia* and HHV6A (A) or HHV6B (B) for different time intervals in the absence (-PenG) or presence of 10 U/ml of Penicillin G (+PenG). Chlamydial DNA was quantified by qPCR using a primer set against chlamydial ORF LcrH/SycD and normalization against 5S rDNA. Data represent the mean ± SEM of three independent infection experiments. (**C,D**) CHO cells do not permit HHV6-mediated chlamydial persistence. Infectivity assays were performed in CHO cells using both HHV6A and HHV6B and analyzed by Western blot (C) or inclusion counting after staining the chlamydial inclusions with an antibody against cHsp60 and Cy2-labeled secondary antibody (D). Statistical analysis was based on the Student t-test, and p>0.05 was considered as insignificant. IFU, inclusion forming units. (**E,F**) Silencing of CD46 prevents HHV6A-mediated chlamydial persistence, but not that of HHV6B. Human CD46 was silenced by transfection of siRNAs in HeLa cells and the knock down efficiency was checked with an antibody against human CD46. Infectivity assays were performed and analyzed by Western blot (E) or inclusion counting (F) after staining the chlamydial inclusions with an antibody against cHsp60 and Cy2 labeled secondary antibody. Statistical analysis was based on the Student t-test, and p>0.05 was considered as insignificant whereas p<0.05 was considered as significant. IFU/ml in figure (D) and (F) represent the mean ± SEM of three independent infection experiments. (**G**) Productive and latent HHV6 infection affects chlamydial replication in HUVEC cells. *C. trachomatis* (Ctr) and HHV6A were used to infect either HUVEC cells, which were or were not pre-infected with HHV6A for 2–3 weeks (latH6A) as indicated. DNA was extracted and used for amplifying bacterial DNA as described under (A). Data represent the mean of 3 independent experiments. hpi, hours post infection; dpi, days post infection. (**H**) Productive HHV6 infection induces chlamydial persistence. Chlamydial infectivity assays were performed and evaluated by detecting chlamydial Hsp60 (cHsp60) by immunoblotting. Fold change values indicate the ratio of cHsp60 to actin values obtained after signal quantification using densitometric analysis. PI, primary infection; SI, secondary infection. NI: no infection.

We then compared genome replication patterns of *C. trachomatis* induced to persistence either by HHV6 co-infection or antibiotics treatment. In general, bacterial replication was higher in HHV6A than HHV6B co-infection (Figure 2A,B), as was measured by quantitative real time PCR (qPCR) of the chlamydial type III secretion chaperone LcrH/SycD. In the presence of penicillin G, a well-known inducer of chlamydial persistence [17], replication of chlamydial DNA stopped at 3–4 days p.i. Chlamydial genome replication continued until day 9 p.i. in both, HHV6A/B co-infections (Figure 2A,B), indicating a continuous replication of chlamydial DNA irrespective of the complete loss of infectivity.

### Chlamydial Persistence Requires Interaction of Viral Particles with Host Cells

To understand the effect of viral activity on bacterial persistence, co-infection experiments with UV-inactivated HHV6A/B strains were performed. The inactivation efficiency was verified by testing the ability of these viruses to initiate productive infection in HSB2 or Molt-3 cells (data not shown). Interestingly, as previously observed during HSV2 co-infection [8], chlamydial persistence was induced with both HHV6 strains after UV inactivation (Figure 1C). This could be explained by the viral capability to enter into the host cell in spite of UV treatment, as we still detected low levels of viral immediate early (IE) gene U94 transcription in HeLa cells infected with UV-inactivated virus (data not shown). We then created HeLa cell lines harboring latent HHV6A or HHV6B DNA by repeated infection of these cells with HHV6A or −6B (see Material and Methods S1, Figure S2B) over a time period of several weeks after which these cells carried 50–100 copies of viral DNA per 1000 cells (data not shown). In addition to this, viral latency was characterized by high expression of latency specific U94 transcripts (Figure S2B) in the absence of any other viral transcripts [18], [19]. Interestingly, chlamydial persistence was not observed in cells latently infected with HHV6A/B strains (Figure 1B,C). Three additional T cell lines containing chromosomally integrated HHV6A (CiHHV6A) (1–2 copies per cell) were infected with *Chlamydia* and were studied for bacterial persistence by transmission electron microscopy (TEM). No persistent inclusions were observed in these cells even after 48 h p.i. (data not shown). Infectivity could not be quantified in these cells since corresponding control cell lines lacking latent HHV6A were not available. Thus our data suggests that direct interaction of HHV6 with host cells is required to induce chlamydial persistence. To check this possibility, CHO cells, which are not susceptible to HHV6A/B, were co-infected and chlamydial infectivity was tested. Neither HHV6A nor HHV6B mediated chlamydial persistence in CHO cells (Figure 2C,D). In line with these findings, RNAi-mediated silencing of CD46, a receptor for entry of HHV6A, but not HHV6B [20], prevented HHV6A, but not HHV6B-mediated chlamydial persistence (Figure 2E,F), supporting the notion that entry of HHV6 is essential for inducing chlamydial persistence during co-infection.

### Induction of Persistence is Independent of Productive Virus Infection

HeLa cells are not productively infected by HHV6 as infected cells cannot produce viral glycoproteins or intact viral particles. Since induction of persistence of *Chlamydia* depended on the continuous presence of the virus in HeLa cells, we asked, if these restrictions also exist in primary cells susceptible to HHV6 infection. Human umbilical vein endothelial cells (HUVEC) serve as a natural reservoir for HHV6 allowing active viral particles to survive for prolonged time without productive viral infection [21], [22]. Most HUVEC cells primarily infected with HHV6A expressed viral proteins like gp116 and p41 by 72 hours p.i. (Figure S2C) which then decreased over time. However, viral DNA and proteins were detectable even 3 weeks after virus infection. When these HUVEC cells after 7–8 passages were infected with *Chlamydia*, the development of chlamydial infectious EB was still inhibited (Figure 2G,H). Thus, in susceptible cells, HHV6 infection induces *Chlamydia* persistence even in the absence of active viral infection.

### *Chlamydia* Infection Favors HHV6 Entry and Survival

**Figure 3:**
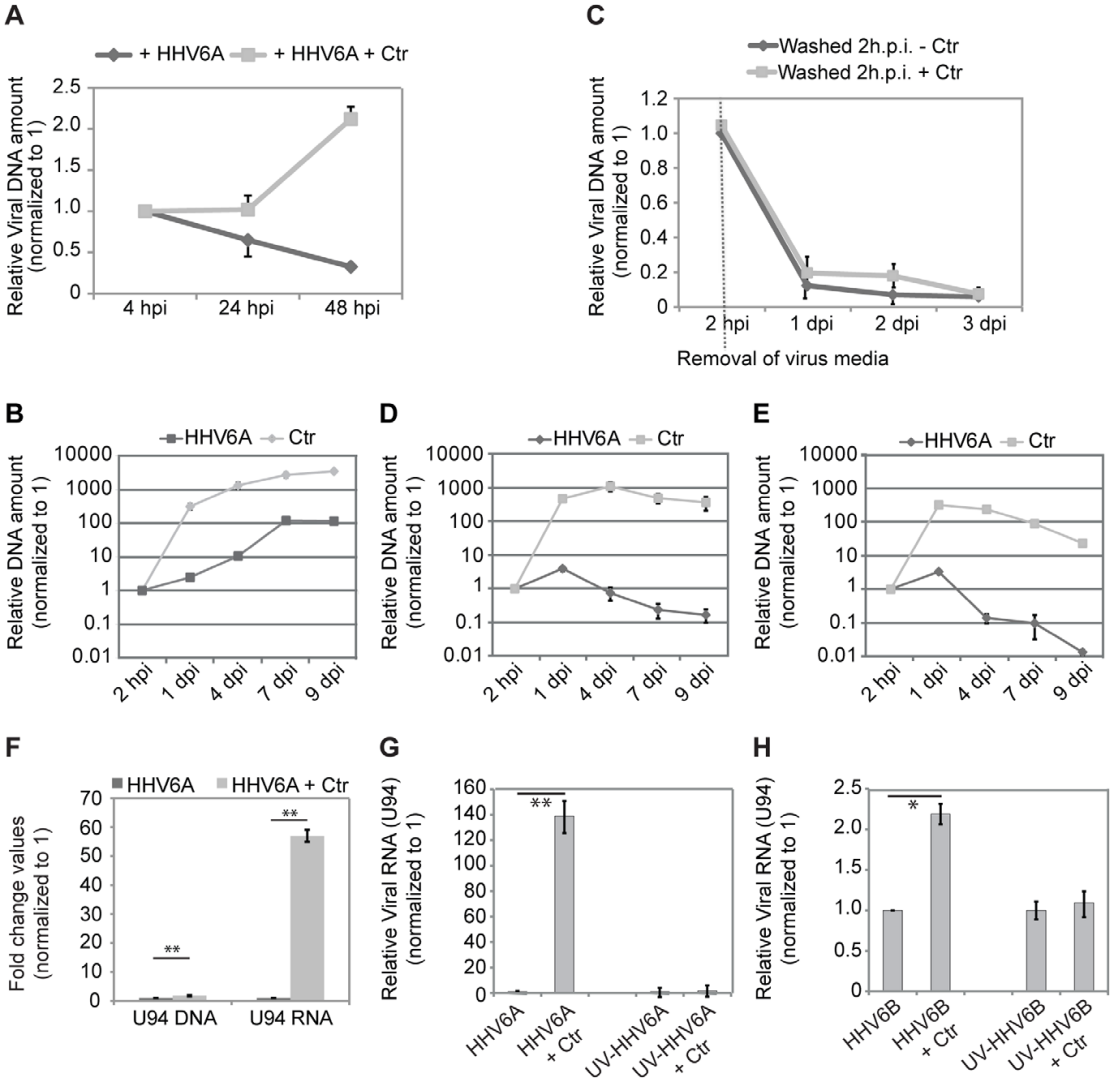
Co-infection of HHV6 and *C. trachomatis* (Ctr) favors viral entry and survival. (**A**) HeLa cells were infected with HHV6A for different time intervals either in presence or absence of Ctr. Viral DNA was quantified by qPCR, using a primer set against viral the U94 gene. (**B**) The amount of HHV6A and *Chlamydia* (Ctr) DNA increases during co-infection. HeLa cells were co-infected with HHV6A and Ctr for different time intervals. DNA was extracted and used for qPCR with primers for viral U94 ORF and chlamydial LcrH/SycD. Relative DNA amount values are presented in log scale. (**C**) Removal of virus particles from the supernatant during co-infection prevents the increase of viral DNA. HeLa cells were infected with HHV6A either in presence or absence of Ctr. Two hours post infection (hpi), cells were washed and fresh culture medium was added. Viral DNA was quantified at different time points by qPCR, using a primer set against viral U94 gene. Data represent the mean of 3 independent experiments. (**D,E**) Inhibition of *Chlamydia* growth with 10 U/ml Penicillin G (B) or 100 ng/ml doxycyclin (C) reduces viral DNA amount. Antibiotics were added to the co-infected cells at 24 h p.i. Data represent the mean of 3 independent experiments. Relative DNA amount values are presented in log scale. hpi, hours post infection; dpi, days post infection. (**F**) Viral U94 transcription is induced during co-infection. HeLa cells were co-infected in duplicates with HHV6A and *Chlamydia* (Ctr) for 24 h. Total DNA and RNA was extracted and viral U94 DNA and RNA transcript levels were quantified by qRT-PCR and normalized against 5S rDNA or 5S rRNA respectively. **, p≤ 0.005. (**G,H**) HHV6A (G) or HHV6B (H) U94 transcription is increased during co-infection. HeLa cells were co-infected with HHV6A, UV-inactivated HHV6A (UV.HHV6A) and *Chlamydia* (Ctr) for 24 h as indicated. Total RNA was extracted and viral U94 transcript levels were quantified by qRT-PCR and normalized against 5S rRNA. hpi, hours post infection; dpi, days post infection. *, p≤ 0.05; **, p≤ 0.005. All the data represent the mean of 3 independent experiments performed on the same day.

The fact that chlamydial infectivity is also affected in cells susceptible to HHV6 infection suggested that induction of persistence rather requires viral products inside a cell than the interaction of the virus with cell surface receptors. We therefore tested the influence of *Chlamydia* infection on viral entry and survival in HeLa cells. Under single infection conditions, HeLa cells do not allow HHV6 survival and replication (Figure 3A). Interestingly, the amount of HHV6 DNA increased dramatically during an active co-infection with *Chlamydia* (Figure 3A, 3B). To ascertain whether the increase in viral DNA results from increased viral entry or replication, we removed the virus containing media 2 h p.i. and washed the co-infected cells thoroughly. This decreased the amount of viral DNA and was comparable to single infections (Figure 3C). Hence, the increase in viral DNA was a result of increased viral uptake rather than viral replication. On the contrary, *Chlamydia* replication was down regulated when co-infected with HHV6 (Figure S3A). We then examined the dependency of increased viral uptake to the presence of active chlamydial growth. For this, we added either Penicillin G or Doxycycline to infected cells at 24 h p.i., which resulted in gradual chlamydial death and/or removal and studied the viral and bacterial DNA amount over a time period of 9 days. Antibiotics treatment did not affect single viral infection (Figure S3B) but strongly reduced the presence of both bacterial and viral DNA in co-infections (Figure 3D,E), suggesting that *Chlamydia* infection significantly supports the entry of HHV6 in non-susceptible cells.

Further, not only viral entry was increased, but also the viral early transcription machinery appeared strongly activated. We compared the viral DNA amount to viral IE gene U94 transcription during HHV6A single infection and co-infection. The viral DNA was 2.5 fold higher after 24 h post infection (Figure 3F), whereas U94 RNA transcript was 55 fold higher under similar infection conditions thus indicating a strong increase in viral transcription. In another set up, transcription of HHV6A and -6B U94 was compared to that of UV-inactivated viruses (Figure 3G,H). Transcription of U94 was dependent on intact viruses since co-infections with UV-inactivated HHV6A and 6B did not result in any major change in U94 transcription (Figure 3G,H). The increase in viral activity was also confirmed by the increased transcription of other IE genes (U42), early genes (U79) and late genes (U22) (Figure S3C). These results suggest that *Chlamydia* infection aids rapid viral uptake and survival inside the host cell accompanied by effective viral gene transcription.

### Co-infection Induces Severe Oxidative Stress

**Figure 4:**
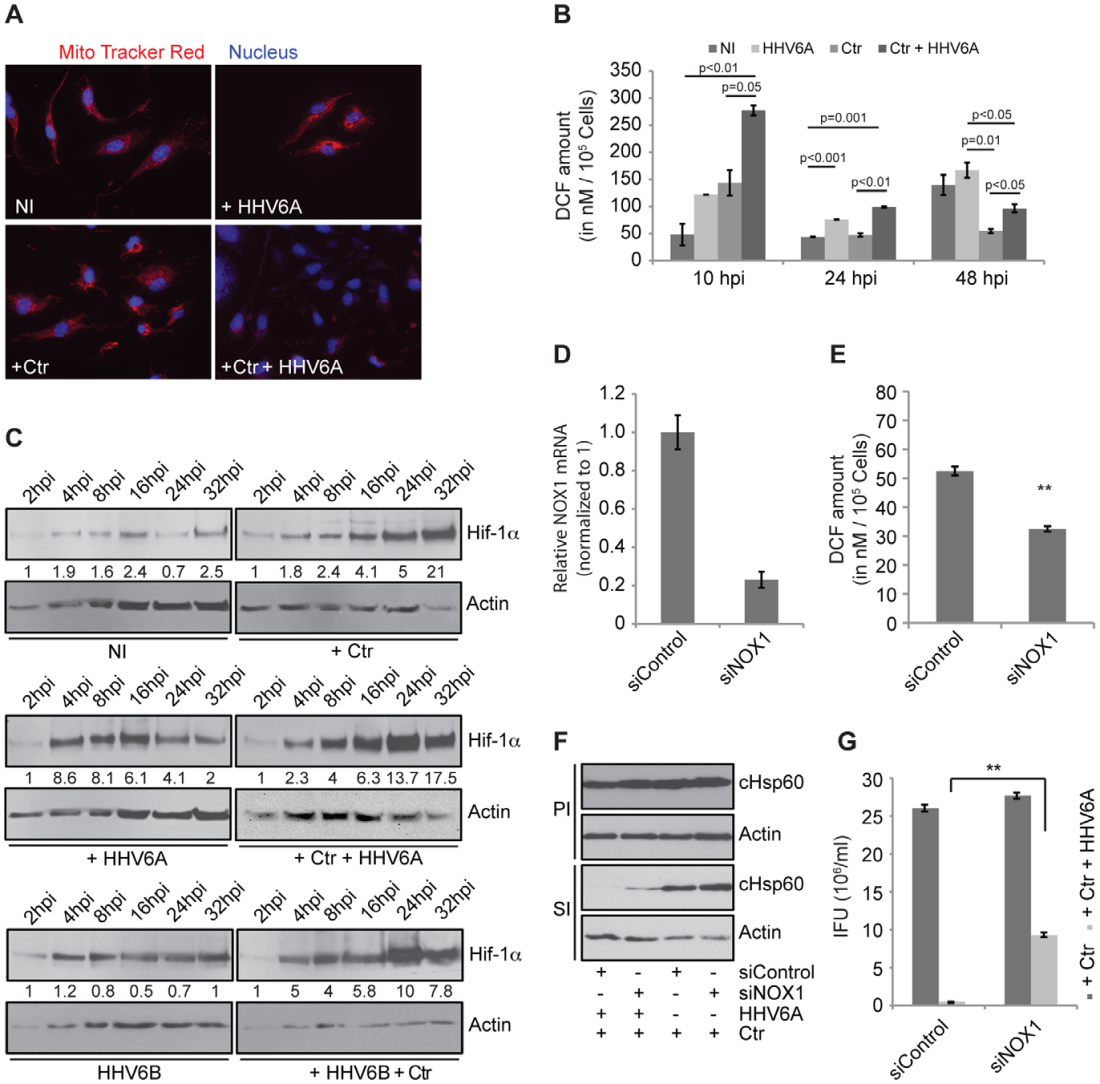
HHV6 co-infection induces mitochondrial membrane potential loss and reactive oxygen species (ROS) formation. (**A**) Co-infection of HHV6 and Ctr down regulates mitochondrial membrane potential in HUVEC cells. HUVEC cells were infected with Ctr and/or HHV6A. Mitochondrial membrane potential was visualized by staining with MitoTracker (red) and fluorescence microscopy. (**B**) HHV6 infection increases ROS in host cells. HeLa cells were infected with *Chlamydia* (Ctr), HHV6A or both for three different time intervals. At the end of the infection, cell-permeable fluorogenic probe 2′, 7′-Dichlorodihydrofluorescin diacetate (DCFH-DA) was added for 1 h at 37°C. Total cellular ROS content was measured using an Elisa reader. Data represent the mean ± SEM of three independent infection experiments performed at the same day. Statistical analysis was based on the Student t-test, and p values between different sample groups are mentioned above the respective line bars. (**C**) HHV6A and -6B induces Hif-1alpha expression. HeLa cells were infected with Ctr and/or HHV6A or -6B for different time intervals and *Chlamydia* (cHSP60) and Hif-1α were detected by immunoblotting. Actin was used as a loading control. Fold change values of Hif-1alpha were derived by normalisation to actin and are mentioned below each lane. (**D**) siRNA-mediated gene silencing efficiency of siNOX1 was checked by quantitative real time PCR. (**E**) Change in cellular ROS content was verified after 24 h of NOX1 silencing. Data represent the mean ± SEM of three independent experiments. **, p≤0.005. (**F, G**) siRNA-mediated gene silencing of NOX1 recovers HHV6-induced persistent *Chlamydia*. HeLa cells were transfected with 5 nM of NOX1 and control siRNA for 48 h and then infected with Ctr alone or together with HHV6A. Infectivity was determined by immunoblotting (**F**) and inclusion counting (**G**). Inclusion counting data represent the mean ± SEM of three independent experiments. **, p≤0.005. PI, primary infection; SI, secondary infection. IFU, inclusion forming units. Data in figure (D), (E) and (G) represent the mean of 3 independent experiments performed on the same day whereas the data in figure (F) represents one of the three biological replicates.

*Chlamydia* growth is directly dependent on host cell ATP levels since they have reduced metabolic activity and depend on the uptake of host nucleotides [23]. Hence, we investigated the integrity of the mitochondria as one source of host cell ATP. We observed a substantial decrease in mitochondrial membrane potential in co-infected HUVEC (Figure 4A) and HeLa cells (Figure S4A). Loss of mitochondrial membrane potential may be the cause or consequence of reactive oxygen species (ROS) [24], [25] that are rapidly converted to H_2_O_2_ by superoxide dismutases (SODs) in the cell. We therefore tested whether infected cells have elevated levels of ROS. In line with recently published data [26], *Chlamydia* infection caused elevated cellular ROS levels at early time points of infection, which decreased to control levels within 24 h of infection (Figure 4B). Co-infected cells showed a similar trend but ROS level remained significantly higher at any time when compared to single *Chlamydia* infection. HHV6 infection induced ROS right from the start of infection and ROS level remained high throughout 48 h of infection (Figure 4B). It is well established that ROS, under normoxic conditions, induces the stabilization of hypoxia-inducible factor 1 alpha (Hif-1alpha) [27], [28], a major transcription factor controlling the physiology and survival of tumor cells. We have recently demonstrated that Hif-1alpha is stabilized in cells infected with *C. trachomatis* and induces the strong up-regulation of the tumor suppressor and apoptosis inhibitor Mcl-1 [29]. Interestingly, co-infection caused a shift in the up-regulation of Hif-1alpha to early time points (4–8 h) and a strongly increased overall expression at later time points (24 h and 32 h) (Figure 4C, S4B, S5). Further, Hif-1alpha was found in the nucleus (Figure S5) and Mcl-1 was highly up-regulated in co-infected cells (Figure S4B), indicative of a full activation of its transcriptional activity. It is well known, that NADPH oxidases are a major source of cellular ROS production [30]. Nox1 is over expressed in many epithelial cell lines and has been linked to mitochondrial ROS generation in response to different stimuli including IFNγ [31]. We therefore tested the involvement of NOX1 in co-infection induced deregulation of ROS production. We silenced the expression of NOX1 (Figure 4D) and tested the effect on chlamydial persistence induced by co-infection. Silencing of NOX1 reduced the co-infection induced production of ROS by 40% (Figure 4E) and rescued chlamydial infectivity to 33% (Figure 4F,G). These results indicated a role of NOX1 induced ROS production for the persistence induced by co-infection.

### GSH Levels are Critical to Maintain Chlamydial Infectivity

**Figure 5:**
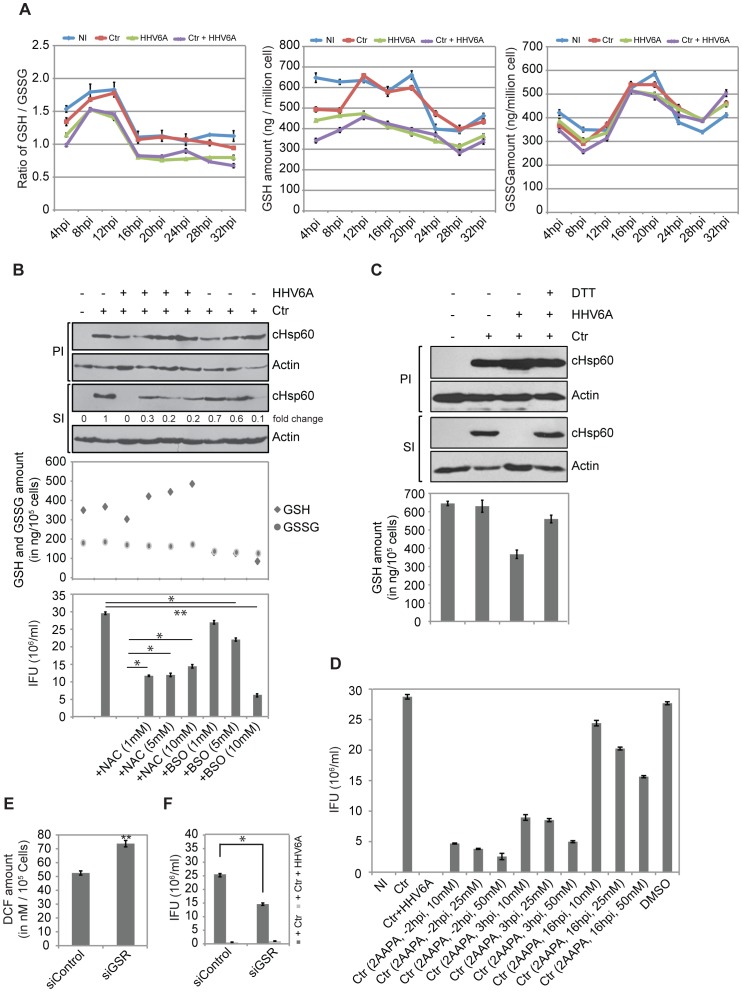
HHV6 co-infection alters cellular glutathione balance. (**A**) Effect of HHV6A and *Chlamydia* infection on cellular GSH and GSSG content. HeLa cells were infected with *Chlamydia* (Ctr) and/or HHV6A for different time intervals. Total GSH and GSSG contents were measured using commercially available kits. Data represent the mean ± SD of three biological replicates performed on the same day. (**B**) Effect of glutathione precursor NAC and inhibitor (BSO) on chlamydial infectivity. HeLa cells were infected with Ctr for 2 h before NAC together with HHV6A or BSO without HHV6A were added at different concentrations. Infectivity assay were performed and analyzed by quantitative immunoblotting and counting of the inclusions. Cellular GSH and GSSG content was measured in parallel sets of infected cells 24 h after addition of NAC and BSO. IFU, inclusion forming units. *, p≤0.05; **, p≤0.005. (**C**) Exogenous supplement of DTT rescues chlamydial infectivity. HeLa cells were infected either with *Chlamydia* (Ctr) or together with HHV6A. Infected cells were supplemented with DTT (1 mM). Infectivity assay were performed to check chlamydial infectivity. The cellular GSH content was measured in parallel sets of primary infected cells 20 h after addition of DTT. PI, primary infection; SI, secondary infection. (**D**) 2-AAPA, an inhibitor of glutathione reductase reduces chlamydial infectivity. 2-AAPA was added to HeLa cells at 3 different concentrations and time points of single (Ctr) infected cells. Co-infection with HHV6A and treatment with the solvent DMSO were used controls. NI, no infection. (**E**) Change in cellular ROS content was measured in HeLa after 24 h of siRNA-mediated GSR silencing. Data represent the mean ± SEM of three independent experiments. **, p≤0.005. (**F**) siRNA-mediated gene silencing of GSR induces Chlamydia persistence. HeLa cells were transfected with 5 nM of GSR siRNA for 48 h. In parallel, a control siRNA pool was also transfected. siRNA transfected cells were then infected with Ctr for 48 h followed by Infectivity assay. Inclusion counting was carried out to check Chlamydial infectivity. Inclusion counting data represent the mean ± SEM of three independent experiments. *, p≤0.05. NI: no infection. Data in figure (D), (E) and (F) represent the mean of 3 independent experiments performed on the same day.

Glutathione peroxidase plays a major role in the detoxification of H_2_O_2_. Reduced glutathione (GSH), a strong antioxidant and free radical scavenger is required for the activity of glutathione peroxidase. Hence, we measured intracellular GSH under different infection conditions over an infection period of 32 h. Interestingly, GSH levels dropped during the early phase of *Chlamydia* growth in single infected compared to non-infected cells but then strongly increased at 12 h p.i. and remained at the level of control cells (Figure 5A). In contrast, GSH level in HHV6A infected as well as co-infected cells were low throughout the time period (Figure 5A), suggesting that the virus counteracts the increase in GSH seen with *Chlamydia* infected cells.

To test the hypothesis, that an imbalance in reduced glutathione (GSH)/oxidized glutathione (GSSG) in the co-infected cell drives *Chlamydia* to persistence, we used reducing agents known to reduce GSSG to GSH. Addition of N-acetyl cysteine (NAC) to co-infected cells indeed increased the GSH levels (Figure 5B). Interestingly, addition of NAC (Figure 5B) partially and of the strong reducing agent Dithiothreitol (DTT) (Figure 5C) completely reverted the persistence of *Chlamydia* in co-infected cells without preventing viral entry (Figure S6A). Furthermore, DTT prevented the early induction of Hif-1alpha and Mcl-1 (Figure S4B), demonstrating a role of oxidative stress in the activation of these important regulators of cell physiology and survival.

In line with these findings, depletion of ROS by the addition of SOD also rescued the chlamydial infectivity (Figure S6B, S6C), demonstrating a general role of oxidative stress in the loss of chlamydial infectivity in co-infections with HHV6. However, in line with an initial increase in ROS (Figure 4B), ROS are required in the early phase of chlamydial infection, since pre-incubation of the cells with SOD completely prevented primary infection (Figure S6B,C).

To test whether interference with the cellular glutathione system is sufficient to cause loss of chlamydial infectivity, we made use of buthionine sulfoximine (BSO), an inhibitor of γ-glutamylcysteine synthetase, which prevents cellular glutathione synthesis. BSO treatment induced a dose-dependent loss of infectivity, but had no effect on the primary infection of *Chlamydia* in the absence of virus infection (Figure 5B), supporting a role of the cellular glutathione system for the development of infectious *Chlamydia*.

### Glutathione Reductase is a Target of Co-infection Induced Chlamydial Persistence

Glutathione reductase (GSR) is the central enzyme that reduces GSSG to GSH. We therefore tested whether interfering with GSR activity could mimic HHV6 infection in inducing chlamydial persistence. Inhibition of GSR activity with 2-acetylamino-3-[4-(2-acetylamino-2-carboxyethylsulfanylthiocarbonyl-amino)phenylthio-carbamoylsulfanyl] propionic acid (2-AAPA) (Figure S6D) induced loss of chlamydial infectivity (Figure 5D). RNAi-induced silencing of host cell GSR (Figure S6E) caused an increase in ROS (Figure 5E) and significantly reduced the formation of infectious *Chlamydia* (Figure 5F, S6F), demonstrating a potential role of GSR for the virus-induced chlamydial persistence.

**Figure 6:**
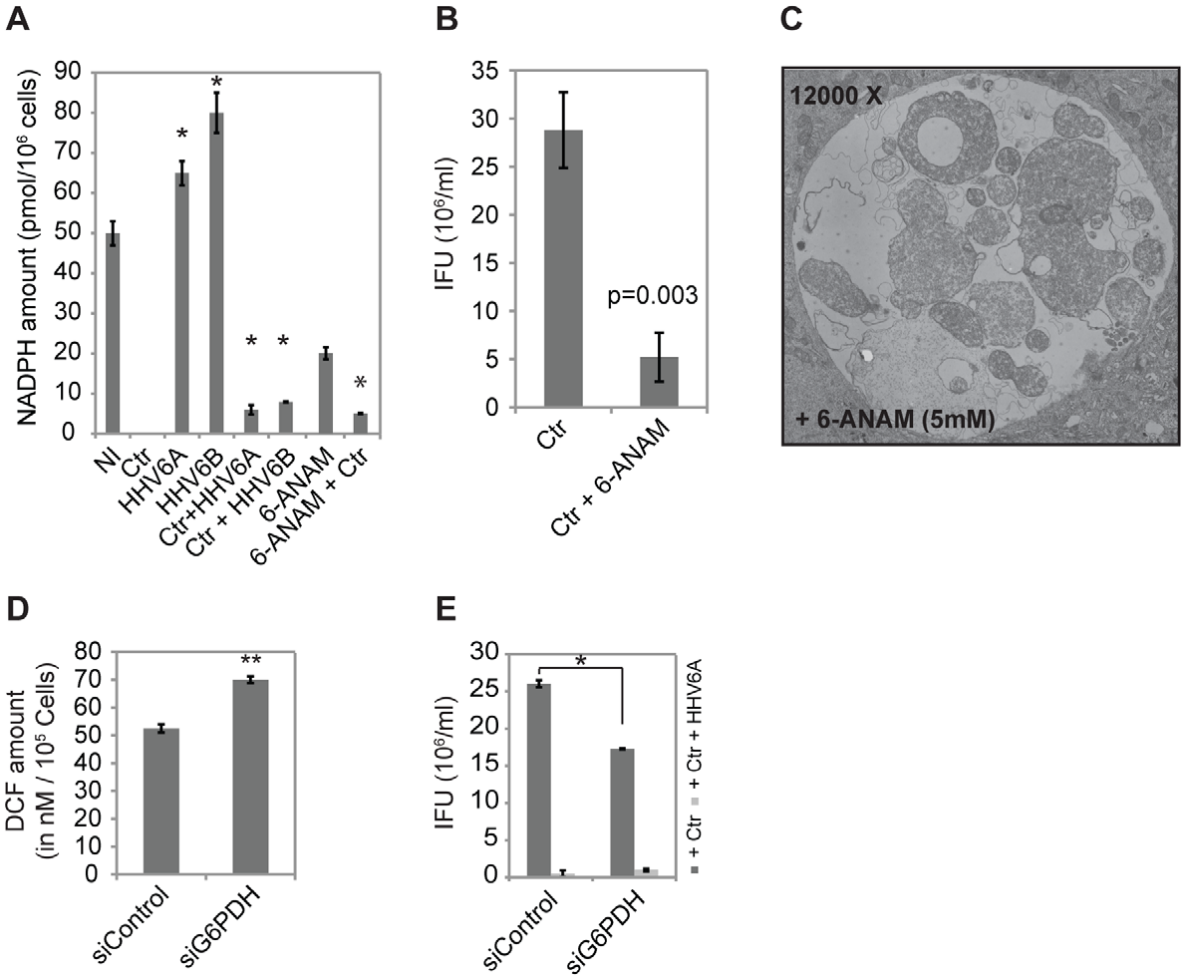
HHV6 co-infection alters cellular NADPH balance by inhibiting GSR activity. (**A**) *Chlamydia* infection depletes, HHV6 infection increases cellular NADPH level. HeLa cells were infected with *Chlamydia* (Ctr) in the presence or absence of HHV6A or HHV6B. Total NADPH content of the cells was measured after 24 h of infection. NADPH level was also measured under similar infection conditions from cells pre-treated with 5 nM 6-ANAM for 24 h. Data represent the mean ± SEM of three independent experiments performed on the same day. *, p≤0.05 (**B**) 6-ANAM induces chlamydial persistency. HeLa cells were treated with 5 nM 6-ANAM for 24 h followed by infectivity assay to measure chlamydial persistency. Inclusion counting was used to check the chlamydial infectivity. Data represent the mean ± SEM of three independent experiments. (**C**) Electron microscopy picture of a chlamydial inclusion in HeLa cells treated with 5 mM 6-ANAM for 24 h. (**D**) Change in cellular ROS content was measured in HeLa cells after 24 h of siRNA-mediated G6PDH silencing. **, p≤0.005. (**E**) siRNA mediated silencing of G6PDH expression reduces *Chlamydia* infectivity. HeLa cells were transfected with 5 nM of G6PDH siRNA for 48 h. In parallel, a control siRNA pool was also transfected. siRNA transfected cells were then infected with Ctr for 48 h followed by Infectivity assay. Inclusion counting was carried out to check chlamydial infectivity. *, p≤0.05. Data in figure (D) and (E) represent the mean ± SEM of three independent experiments performed on the same day.

GSR depends on NADPH as co-enzyme and donor for electrons for the reduction of GSSG to GSH. We therefore measured NADPH levels in infected and co-infected cells, since it is an indicator of the enzyme activity [32]. Surprisingly, NADPH levels were not measurable in *Chlamydia*-infected cells (Figure 6A), suggesting that NADPH is fully consumed in infected cells. Low NADPH levels are in line with high levels of GSH and active detoxification of ROS (Figure 4B, 5A). Increased NADPH concentrations compared to control cells were measured in cells infected with HHV6A or HHV6B, whereas NADPH levels were low but measurable in co-infected cells (Figure 6A), indicating reduced GSR activity in virus- and co-infected cells. In line with NADPH levels as indicators for the GSR reaction, NAC treatment, which rescued chlamydial infectivity (Figure 5B), reduced the increased NADPH levels in co-infected cells (Figure S6G). Consistently, interfering with glutathione biosynthesis by BSO treatment caused a dose-dependent increase in NADPH in *Chlamydia* single infected cells comparable to the co-infected cells (Figure S6E), which interfered with chlamydial infectivity but not primary infection (Figure 5B).

To confirm the central role of the GSR reaction in chlamydial persistence induced by co-infection, we interfered with NADPH synthesis by inhibiting the pentose phosphate pathway (PPP), the central source of NADPH. Inhibition of glucose 6-phosphate dehydrogenase (G6PDH) by 6-aminonicotinamide (6-ANAM) decreased cellular NADPH content (Figure 6A) and induced persistence of *Chlamydia* with all ultra-structure hallmarks of chlamydial inclusions observed under co-infection conditions (Figure 6B,C). To test whether specifically targeting the host PPP causes a similar effect, G6PDH expression was silenced by RNAi. Knockdown of G6PDH expression (Figure S6E) caused an increase in ROS (FFure 6D) and a partial induction of chlamydial persistence in the absence of viral co-infection (Figure 6E, S6F).

In conclusion, the strong decrease in GSH level and elevation of NADPH levels in virus infected cells suggested that HHV6 interferes with the GSR activity provoking an imbalance in the detoxification of ROS and, as a consequence, the induction of chlamydial persistence during co-infection.

### Herpes Simplex Virus (HSV)-1 and Human Cytomegalovirus (HCMV) Induce ROS-dependent Chlamydial Persistence

**Figure 7:**
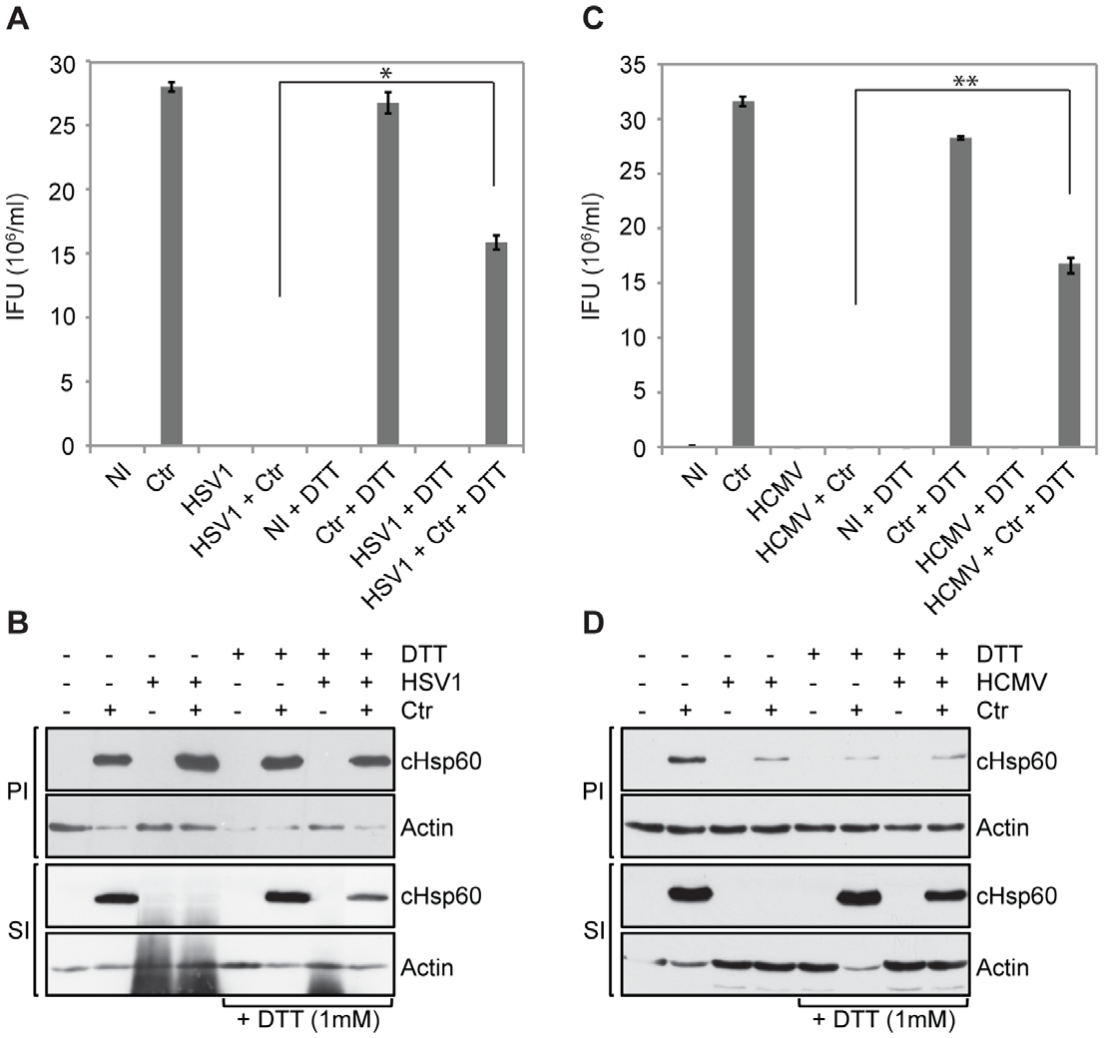
HCMV and HSV-1 induce chlamydial persistence. HeLa cells were infected or co-infected with *Chlamydia* (Ctr) and (**A,B**) HSV-1 or (**C,D**) HCMV. Infectivity was monitored by counting inclusion numbers after staining the chlamydial inclusions with an antibody against cHsp60 and Cy2 labeled secondary antibody (A and C) and immunoblotting (B and D). PI, primary infection; SI, secondary infection; NI: no infection; IFU, inclusion forming units. Data in figure (A) and (C) represent the mean ± SEM of three independent experiments performed on the same day. *, p≤0.05; **, p≤0.005. Figure (B) and (D) represent one of the three biological replicates.

To test whether other herpes viruses also provoke chlamydial persistence by a similar mechanism as HHV6, co-infection with HSV-1 and HCMV, members of the alpha and beta herpes virus group, respectively, was established. Co-infection with HSV-1 and HCMV induced loss of chlamydial infectivity and this effect could be reverted by scavenging ROS with DTT (Figure 7A, B, C, D), demonstrating that the mechanism described here is of broader significance in the interaction of *Chlamydia* and herpes viruses.

## Discussion

*C. trachomatis* is the most frequent obligate intracellular human pathogenic bacterium that infects many different cell types and causes a wide variety of infectious diseases. Here, we show that chlamydial co-infections with HHV6, one of the most prevalent viruses in humans worldwide, massively influence the infectivity of the bacteria by inducing fully reversible persistence. This effect is independent of productive virus infection and largely depends on prolonged oxidative stress in the co-infected cells.

The amazingly strong and complete induction of chlamydial persistence in presence of high HHV6 titers (MOI of 5–10) was special in several aspects. Unlike as in persistence induced by antibiotics treatment, bacteria retained DNA replication for several days but re-entered the productive developmental cycle nearly quantitatively as soon as the virus was removed from the culture in line with the observations of recovery after HSV2-mediated [6] as well as penicillin-induced persistent *Chlamydia* [33], [34]. It is therefore very likely that co-infection induces a distinct persistence program in *Chlamydia* that not only enables the bacteria to rapidly exit from the productive developmental cycle but also to immediately recover once the virus is removed from the cell. HHV6 co-infection had no effect on the infectivity of *Simkania,* which unlike *C. trachomatis* and *C. pneumoniae* can also infect and grow in amoeba [35]. It is therefore tempting to speculate that human specific HHV6 and the human pathogenic *Chlamydia* co-evolved in or compete for related niches.

Blood cells are suspected to act as a host and carrier of *C. trachomatis* as well as *C. pneumoniae* as infectious *Chlamydia* have been detected in patient blood samples [36], [37]. Although HHV6 infects both T-cells as well as macrophages, it has a productive life cycle in T-cells but achieves latency in other cell types including macrophages [38]. Interestingly, HHV6 can still maintain the replicative life cycle for several days in macrophages and can also be re-activated from latency in these cells by different factors including phorbol ester [38]. Thus, human peripheral blood cells are both natural targets for *C. trachomatis* and active HHV6 infection and thus represent prime sites for co-infection of both these pathogens *in vivo*. *C. trachomatis* maintains a productive infection in monocyte-derived macrophages [16] and in human macrophages from healthy blood donors (Figure 1E). However, macrophages co-infected with HHV6A and *C. trachomatis* contained mostly persistent *Chlamydia* similarly as in epithelial and endothelial cells. Interestingly, although the blood cell infection procedure allowed viral and chlamydial co-infection only for one hour, subsequent removal of extracellular virus and *Chlamydia* from culture media by stringent washing did not prevent occurrence of persistent *Chlamydia* infections for as long as 72 h in these cells. This observation is in cohorts with our data from HUVEC cells. Hence, the possibility of natural co-infections of HHV6 and *C. trachomatis* is indisputable.

The competition for similar niches may be a more general phenomenon in the interaction of viruses and *Chlamydia.* HCMV, HSV-1 (Figure 7) and HSV-2 [6], [7], [8], [9] have been shown to induce persistence in *C. trachomatis* co-infections with similar features as those described here for HHV6. HSV-1 and -2 (alpha-herpesvirus) differ in their host cell specificity from HCMV and HHV6 (beta- herpesvirus). The viral preference for lytic growth or latency also depends on the cell type. How these viruses despite of their individual differences similarly affect chlamydial growth is an open question. It has been shown that HSV2 glycoprotein D induces chlamydial persistency [6]. We could not find sequence homology between HSV2 glycoprotein D with any of the HHV6 glycoproteins. In addition, we tested the effect of HHV6 glycoprotein Q1, Q2, B and H on chlamydial growth and could not observe any significant effect (data not shown), suggesting the involvement of a completely different signaling pathway capable of altering bacterial growth and infectivity. Further, co-infections of *C. pecorum* with porcine epidemic diarrhea virus (PEDV) induced persistence in Vero cells [39], [40]. Our data on the rescue of chlamydial infectivity by applying ROS scavengers and reducing agents to cells co-infected with HHV6A, HHV6B, HCMV and HSV-1 indicated a similar and general mechanism driving *Chlamydia* into persistence in these cells. It may be possible that different herpes viruses have evolved additional or independent mechanisms to regulate host cell physiology, which can interfere with chlamydial growth.

We have used multiple approaches to show that HHV6 entry into the host cell is essential for the development of chlamydial persistency. Absence (Figure 2C, D) or RNAi mediated knock down (Figure 2E, F) of HHV6A-specific receptor, CD46, decreased HHV6A-mediated chlamydial persistency. Although HHV6 is a lymphotropic virus that replicates in CD4+ or CD8+ T lymphocytes, the HHV6A receptor CD46 required for virus entry is present on all nucleated cells [41]. Our data showing that only uptake of the virus, but not the formation of virus particles as a prerequisite for induction of chlamydial persistence is a key finding since it implicates that the virus and *Chlamydia* can interact in any cell, which can be infected by both pathogens. The dramatically increased uptake of virus into *Chlamydia*-infected cells argues in favor for a close interaction of both pathogens already at this critical point of the infection process. Non-productive virus infection, on the other side, is not a prerequisite for its interference with chlamydial infectivity since productively HHV6-infected primary HUVECs push *Chlamydia* into persistence as well. Thus, a pre-existing chlamydial infection strongly sensitizes cells for HHV6 infection, which in turn causes the temporal exit of the bacteria from the developmental cycle and co-existence in cells either susceptible or insusceptible for virus production.

Production of ROS is a general mechanism of cells to defeat viruses and bacteria [42]. We found that infection of HeLa cells with *C. trachomatis* alone induces transient ROS production confirming recently published data [26], [43]. The production of ROS has been suggested to be initially induced by the activation of the NADPH oxidase which then is rapidly inactivated in infected cells, possibly by the recruitment of the regulatory subunit to the inclusion Rac1 [26]. Inhibition of the bacterial protein synthesis or the chlamydial Type 3 Secretion System prevented the induction of ROS, supporting the idea of an active role of *Chlamydia* in the induction of ROS as well. This transient activation of ROS during chlamydial infection may initiate signaling cascades like the activation of caspase-1 [43] and Hif-1alpha [29], which are beneficial for the bacterial development. But these signaling cascades strongly activated during co-infection may also influence the physiology of the host cell. Hif-1alpha is one of the most important transcription factors regulating the glycolytic metabolism and *de novo* fatty acid synthesis in tumor cells [44], [45]. This change in metabolism, also referred to as Warburg effect, is induced by Herpes viruses like CMV and Kaposi’s sarcoma herpes virus to support the production of viral particles [46]. The high levels of ROS, Hif-1alpha and Mcl-1 strongly suggest that modified signaling in co-infected cells may, in addition, support pathologies like cancer development and neurodegeneration [47], [48].

But how could imbalanced ROS production in co-infected cells affect the development of *Chlamydia*? The high structural rigidity of chlamydial EB is caused by the highly cross-linked outer membrane protein complex, which is brought about by the intra- and intermolecular cysteine bonds in the cysteine-rich proteins of the EB outer envelope, such as OmpA, OmcB, and OmcA. Early work by the Hackstadt group has already postulated that reduction of outer membrane protein complex proteins is a prerequisite for chlamydial EB to RB transition [49]. Recently, GSH has been suggested to be the natural agent responsible for the reduction of the outer membrane proteins of *C. trachomatis* during EB to RB transition [50]. We observed decreased chlamydial infectivity due to decrease in cellular GSH during later time points of *Chlamydia* infection indicating a more important role of GSH for chlamydial infectivity. Formation of infectious EB is dependent on availability of several cysteine-rich proteins including MOMP, CrpA, OmpA, and OmcB [51], [52], which are incorporated either in a monomeric or cross-linked form to the outer membrane of newly formed EBs. Disulfide bond formation is crucial for the structure and stability of chlamydial proteins including MOMP whose disulfide bonds have been shown to be dynamic rather than stable and adapt to structural changes during the RB and EB transition [53]. Accumulation of NADPH together with decreased GSH during co-infection could influence the reduction of disulfide bonds of these proteins and thus may affect RB to EB re-differentiation.

The conversion of GSSG to GSH involves NADPH and the key enzyme GSR. Consistent with increased consumption of GSH by *Chlamydia*, single infected cells were virtually depleted of NADPH (Figure 6A) and the GSH levels, which were reduced at early time points, increased for the period of the infection cycle when EB transform to RB (8–20 h; Figure 5A). Furthermore, only reducing agents, which increased the GSH level, also rescued chlamydial infectivity in co-infected cells. And finally, interfering with GSH production either indirectly by depleting NADPH or directly by inhibiting GSH synthesis induced chlamydial persistence reminiscent to the co-infection situation. We therefore hypothesize that HHV6 co-infection directly interferes with the initial conversion of RB to EB by generating and maintaining an imbalanced GSH/GSSG ratio. This model would also explain why addition of HHV6 virus particles after RB to EB conversion at around 24 h post chlamydial infection did not affect chlamydial persistence (Figure 1D), since GSH levels are similar around this time point in single and co-infected cells (Figure 5A). Bactericidal antibiotics including Penicillin G decreases NADPH level in cell either by modulating G6PDH function [54] or by stimulation of oxidation of NADH leading to depletion of reducing equivalents with concomitant increase in hydrogen radical mediated damage [55]. Decreased NADPH level could alter the cellular GSH balance, which can be a possible cause for penicillin-induced chlamydial persistency. These observations support our results and points towards common machinery that can be crucial for the successful transition of chlamydial RB to EB development.

Decrease in GSH level and concomitant elevation of NADPH levels in the virus single infected cells strongly suggests that HHV6 infection interferes with GSR activity and thus causes an imbalance in the detoxification of ROS. Our findings of a role of ROS and GSH levels in the competition between virus and *Chlamydia* provide a first insight into the mechanism by which chlamydial persistence is induced in these co-infections. We are just beginning to understand how the co-evolution of one of the most prevalent human pathogenic bacteria and viruses has affected their survival and replication strategies, but also their infective properties while occupying similar niches inside the cell. The *Chlamydia* - HHV6 interaction may thus provide a paradigm for a so far unrecognized close interaction of obligate intracellular bacteria and viruses.

## Materials and Methods

### Cell Culture and Virus Stock Preparation

HSB2 cells and HHV6A viral particles were kindly provided by Prof. Harald zur Hausen [56]. HUVEC cells were purchased from ENZO life sciences, Germany. Molt3 cells, CiHHV6A cell lines and HHV6B (Z29) viral particles were kindly provided by the HHV6 foundation, USA. HeLa, HSB2, Molt3 and CiHHV6A cells were grown in RPMI 1640 media and 5% fetal bovine serum (FBS) at 37°C and 5% CO_2_. Primary HUVEC were grown in endothelial cell growth medium (Promo Cell) with added growth additives (C-39215, Promo Cell). HHV6A and HHV6B were infected and propagated in HSB2 and Molt3 cells respectively without any antibiotics (see Material and Methods S1).

### PBMC Isolation

Fresh PBMCs were first separated from whole blood using Histopaq1077 solution (Sigma, St. Louis, MO, USA) using manufacturer’s instructions. Briefly, freshly collected blood was diluted 2 times with PBS and layered carefully onto 1 volume of Histopaq1077 solution in a falcon tube without mixing the solutions. PBMCs were collected after centrifugation at 600×g for 30 min at room temperature without applying brake. Cells were washed 3 times with 25 volumes of PBS and were kept in RPMI 1640 media with 5% fetal bovine serum (FBS) and 5 µg/ml of phytohemagglutinin (PHA) (Sigma, USA) at 37°C and 5% CO_2_. PHA stimulation was used to induce HHV6 infection in PBMCs [38]. Isolated PBMCs were cultured on a plastic culture disc (NUNC, USA) for 48 h to separate monocytes from rest of the blood cells. Separated adherent monocytes were further cultured for 5–7 days in presence of 50 ng/ml macrophage colony stimulating factor (M-CSF) (Sigma, USA) to allow complete differentiation into macrophages. Both the adherent macrophage fraction and the PBMC fractions were infected with Chlymdia and/or HHV6 separately.

### *Chlamydia* Infection and Co-infection

The preparation of chlamydial stocks has been described previously [57]. One day before infection, epithelial cells were seeded in 6 or 12-well plates (Greiner) to reach sub-confluency. Cell media was replaced by fresh medium (RPMI 1640, 5% FBS) containing *Chlamydia* or were left uninfected (control). Cells were incubated at 35°C and 5% CO_2_ for 2.5 h before the medium was replaced by fresh cell culture medium for further incubation.

HSB2 cells, CiHHV6A cells and PBMCs (both monocyte derived macrophages and leukocytes) were infected with *C. trachomatis* at an MOI of 2 either in an Eppendorf tube or on a culture plate and were centrifuged for 1 h at 37°C at 1200g (modified from Manor and Sarov, 1986 [16]). Cells were washed 3 times with PBS and were grown in fresh RPMI 1640 media with 5% FBS.

For all co-infection experiments, both HHV6 and *Chlamydia* were added to the cells at the same time unless otherwise mentioned. For some of the experiments, viral particles were inactivated by UV exposure (2×10^6^ µJ) for 2 h (UV.HHV6A and UV.HHV6B) prior to co-infection and were added simultaneously with *Chlamydia*.

### Infectivity Assay

48 h p.i., cells (infected or uninfected controls) were washed once with PBS and lysed by a freeze-thaw cycle followed by pipetting the cells repeatedly. 1:100 diluted cell lysates containing EB were transferred to uninfected cells that had been seeded into 24 wells on glass slides the day before, incubated for 24 h at 35°C and fixed in 4% formaldehyde (PFA). After permeabilization with PBS +0.2% Triton X-100 for 20 min at RT and blocking with PBS +10% FBS for 1 h at RT, cells were stained with an antibody against chlamydial Hsp60 (cHsp60) in PBS +2% FBS at RT for 1 h. Cells were washed with PBS thrice and incubated with a Cy2-labeled secondary antibody and Hoechst (or DAPI) in PBS +2% FBS for 1 h in the dark at RT. Slides were washed twice in PBS, once in distilled water to remove PBS and were mounted on Mowiol (Carl Roth). The number of inclusions was determined by counting ten random fields using epifluorescence microscope (Leica) at 40x magnification. For confocal laser scanning microscopy, samples were stained with Draq5 instead of Hoechst/Dapi and analyzed using a Leica TCS SPE equipped with 488, 532, and 635 nm solid-state lasers for excitation. Images were taken using appropriate excitation and emission filters for the fluorescence dyes. Overlay images of the single channels were obtained using ImageJ. Infectivity was also determined by quantifying chlamydial Hsp60 (cHsp60) by immunoblotting using antibodies against Chlamydia specific Hsp60. The values were normalized to actin after signal quantification using densitometric analysis and ImageJ software. Number of inclusion forming units (IFU) per ml was determined by analyzing five random fields in an epifluorescence microscope with 400-fold magnification.

### Immunoblotting

Cell lysates were resolved by 10% sodium dodecyl sulfate (SDS)-poly- acrylamide gel electrophoresis. Proteins were transferred to polyvinylidene difluoride membranes (Millipore) and blocked with 10% skimmed milk. The membranes were then probed with respective primary antibodies and subsequently with HRP-conjugated secondary antibodies. Proteins were detected with peroxidase-coupled secondary antibody using the ECL system (Amersham). Antisera and antibodies used in this study are listed under materials and methods S1.

### Cell Transfections and RNA Interference

Transfections were performed using Lipofectamine 2000 (Invitrogen) as per manufacturer’s protocol. To silence CD46, G6PDH, GSR, Nox1 gene expression by RNA interference, 1×10^5^ cells per well were seeded into a 12-well plate on the day of transfection. Freshly seeded HeLa cells were transfected with 160 nM siRNAs using RNAiFect transfection reagent (QIAGEN, Valencia, CA) according to the manufacturer’s instructions. Efficiency of gene silencing was generally validated either by western blot analysis or qPCR at 72 h post-transfection. All the siRNAs (ON-TARGETplus smartpool) including a control siRNA pool were purchased from Dharmacon, Thermo Fisher Scientific.

### NADPH Measurement

Total cellular NADPH level measured by NADP/NADPH Assay Kit (Abcam) using manufacturers protocol. To detect NADPH only, NADP was decomposed before the final reaction by heating the samples at 60°C for 30 min in a heating block. Bioluminescence was measured on an ELISA reader (TECAN Infinite M200) at 450 nM.

### Thiol Fluorescence Assay for GSH Measurement

Infected cells were washed three times with warm PBS. Cells were lysed by three freeze–thaw cycles at -80°C. Thiol-reactive Alexa Fluor 488 C5 maleimide (Invitrogen, USA) was added to the cell lysate at a dilution of 1:5000 (stock 10 µg/µl). After an incubation of 5–10 min, the GSH content in the cell lysates were measured by an ELISA plate reader (TECAN Infinite M200) using an excitation filter of 480 nm and emission filter of 530 nm.

### DCF Assay for Cellular ROS Activity Study

Total cellular ROS activity was measured using OxiSelect™ Intracellular ROS Assay Kit (Cell Biolabs Inc., USA) according to the manufacturer’s protocol and an ELISA plate reader (TECAN Infinite M200) with an excitation filter of 480 nm and emission filter of 530 nm.

### Statistical Analysis

Statistical significance (p values) of acquired data was calculated using two-sided Student’s t-test.

## Supporting Information

Figure S1 **(A,B)** HeLa cells were co-infected with HHV6A and 6B and *C. pneumoniae* (A) or HHV6A and *S. negevensis* (B) and infectivity was determined after 3 or 4 days post infection, respectively. Cell supernatants were collected 72 h p.i. and were added to freshly growing HeLa cells. At 24 h p.i., cells were fixed and stained for bacterial Hsp60 protein with an antibody against cHsp60 and Cy2-coupled secondary antibody. Inclusion numbers were counted under a fluorescence microscope against the nuclear DAPI staining. IFU, inclusion forming units. Data represent the mean ± SEM of two independent experiments. **(C)** Early co-infection with HHV6 is necessary for inducing chlamydial persistence. HeLa cells were infected with *Chlamydia* for 2 h prior to the addition of viral particles for different time points as indicated. In a parallel infection set up, HHV6A was added to *Chlamydia-*infected cells after 2 h, but subsequently HHV6 was removed from the infection media at the indicated time points. Infectivity assays were performed as described in Material and Methods and immunoblotting was done to check the bacterial Hsp60 protein (cHsp60) expression. PI, primary infection; SI, secondary infection; IFU, inclusion forming units.
(TIF)

Figure S2 **(A)** Interferon response is unchanged in single and co-infected cells. Heatmap analysis of microarray data showing differentially expressed interferon genes in HeLa cells under different infection conditions as compared to non-infected cells. Total RNA preparations from non-infected as well as HeLa cells infected with *C. trachomatis* (Ctr) and/or HHV6B were analyzed. White or red colors indicate differentially up- or down-regulated genes, respectively according to their log2 fold change values. IFNG, Interferon gamma; IFNE, Interferon epsilon; IFNB1, Interferon betta 1; IFNK, Interferon kappa; IL28A, Interleukin 28A; IFNA8, Interferon alpha 8; IFNA4, Interferon alpha 4; IFNA10, Interferon alpha 10; IFNA16, Interferon alpha 16; IFNA2, Interferon alpha 2; MX1, myxovirus resistance 1; IL18, Interleukin 18; IFNA6, Interferon alpha 6; IL29, Interleukin 29; IL6, Interleukin 6; IL28B, Interleukin 28B; IFNA21, Interferon alpha 21. **(B)** High HHV6 U94 transcription together with the lack of other viral gene transcription demonstrates viral latency. Viral latency was characterized by amplification of HHV6 U94 and U22 transcripts. High transcription of U94 together with absence of U22 transcripts validated that the HHV6 genome was maintained in a latent state. **(C)** HHV6 glycoproteins are expressed in infected HUVEC cells. HUVEC cells were infected with HHV6A for 72 h and gp116 and p41 were detected by immunostaining using antibodies against the respective proteins and Cy3-coupled secondary antibodies. Draq5 staining was used to stain cellular DNA. Samples were viewed under a confocal laser microscopy. Scale bar, 10 µm.
(TIF)

Figure S3 Co-infection of HHV6A and *C. trachomatis* (Ctr) favors viral survival and entry. **(A)** Chlamydial replication is down regulated by HHV6. HeLa cells were infected with *C. trachomatis* and/or HHV6A for different time intervals and DNA was extracted from these cells. Chlamydial DNA was quantified by qPCR, using a primer set against chlamydial LcrH/SycD. Relative viral and Ctr DNA quantity were derived by normalizing the values against 5S rDNA as internal control. In all the graphs, relative values are normalized to 1. Data represent the mean ± SEM of three independent experiments. **(B)** Penicillin G and Doxycyclin have no effect on single infection with HHV6A. HSB2 cells were infected with HHV6A in absence of any antibiotics. In parallel 2 other sets of HSB2 cells were infected with HHV6A either in presence of 10 U/ml of Penicillin G or 100 ng/ml of Doxycyclin for different times. DNA was extracted and used for qPCR with primers against viral U94 ORF. Relative quantity of U94 level was derived by normalizing against 5s rDNA as internal control. Relative viral DNA values are normalized to 1. Data represent the mean ± SEM of three independent experiments. hpi, hours post infection; dpi, days post infection. **(C)** HHV6B gene transcription is induced during co-infection with Ctr. HeLa cells were either infected with HHV6A or Ctr alone or co-infected together for 24 h. Total RNA was extracted, reverse transcribed and used for semi-quantitative RT-PCR using primers against viral U22, U42, U79, U91 and U94 ORFs. Amplified products were run on a 2% agarose gel. GAPDH amplification was used as an internal control. Fold change values were derived by dividing respective band intensity with GAPDH band intensity and are mentioned below respective bands. RT, reverse transcriptase; M, marker.
(TIF)

Figure S4 **(A)** Co-infection of HHV6 and Ctr down regulates host cell mitochondrial membrane potential and induces cytochrome c release. HeLa cells were infected with Ctr and/or HHV6A. As a control, persistence was induced with penicillin G (PenG). Mitochondrial membrane potential was measured in HeLa cells by staining with Mito Tracker (red). Co-staining was done for cytochrome c (green) with using antibody against human cytochrome c and Cy2-coupled secondary antibody (green). Co-localization of mitochondria and cytochrome c was studied under a confocal microscope. **(B)** HHV-6A and -6B induces Hif-1alpha. HeLa cells were infected with Ctr and/or HHV-6A for different time intervals either in the presence (+DTT) or absence (-DTT) of 1 mM DTT. *Chlamydia* (cHSP60), Hif-1α and Mcl-1 expression was detected by immunoblotting. Actin was used as a loading control. Fold change values of Hif-1alpha and Mcl-1 was derived by dividing respective values with Actin and are mentioned below each lane.
(TIF)

Figure S5 Hif-1α expression is induced during co-infection with HHV6. HeLa cells were infected with Ctr and/or HHV6A for different times. Cells were fixed and stained for Hif-1alpha using an antibody against human Hif-1alpha and Cy3-coupled secondary antibody (Red). Host cell DNA was stained with DAPI (blue). Fluorescence microscopy was used to visualize the localization of Hif-1α.
(TIF)

Figure S6 **(A)** DTT has no negative effect on HHV6 survival inside the host cell during co-infection. HeLa cells were infected either with Ctr or together with HHV6A. Infected cells were supplemented with DTT (1 mM). Total RNA was extracted after 24 h of infection and viral U94 transcript level was quantitated using primers against HHV6A U94. Relative U94 transcript level was derived by normalizing the values against 5s rRNA internal control. Relative U94 values are normalized to 1. Data represent the mean ± SEM of three independent experiments. **(B & C)** SOD prevents HHV6-mediated chlamydial persistence. HeLa cells were either first treated with SOD (−2 h p.i.) or directly infected either with *Chlamydia* (Ctr) or together with HHV6A. SOD at different concentrations was added to cells at 3 different time points of infection. Infectivity assay was performed as described in material and methods to check chlamydial infectivity. Immunoblotting (B) was done and inclusion numbers were counted (C) to check chlamydial infectivity. IFU, inclusion forming units. Immunoblot (B) represents one of the three biological replicates. IFU/ml data (C) represent the mean ± SEM of three independent experiments. **(D)** Total cellular GSH and GSSG level was measured in HeLa cells after 24 hrs of 2-AAPA treatment. Data represent the mean ± SEM of three independent experiments. **(E)** siRNA mediated gene silencing of G6PDH, GSR was verified by immunoblotting. Data represents one of the three biological replicates. **(F)** siRNA mediated gene silencing of G6PDH, GSR decreases Chlamydial infectivity. HeLa cells were transfected with 5 nM of G6PDH, GSR siRNAs for 48 hrs. In parallel, a control siRNA pool was also transfected. siRNA transfected cells were then infected with Ctr or together with HHV6A. Infectivity assay and immunoblotting was carried out to check Chlamydial infectivity. siRNA-mediated gene silencing efficiency was checked by using antibodies against G6PDH and GSR. Actin was detected as a loading control. Data represents one of the three biological replicates. **(G)** N-acetyl-l-cysteine (NAC) and buthionine sulfoximine (BSO) changes cellular NADPH level. HeLa cells were infected with *C. trachomatis* (Ctr) alone or together with HHV6A. Infected cells were treated with NAC or BSO at 3 different concentrations for 24 h. Total NADPH content of the cells was measured after 24 h of infection. Data presented here are the mean of 3 independent experiments. PI, primary infection; SI, secondary infection; NI: no infection.
(TIF)

Table S1 Primers used for semi-quantitative RT-PCR and qRT-PCR.
(DOC)

Materials and Methods S1. (DOCX)
