# Supplementary figures and images for: Imbalanced Oxidative Stress Causes Chlamydial Persistence during Non-Productive Human Herpes Virus Co-Infection
Source: PLoS One. 2012 Oct 15;7(10):e47427. doi: 10.1371/journal.pone.0047427 (PMC3471814; doi:10.1371/journal.pone.0047427)

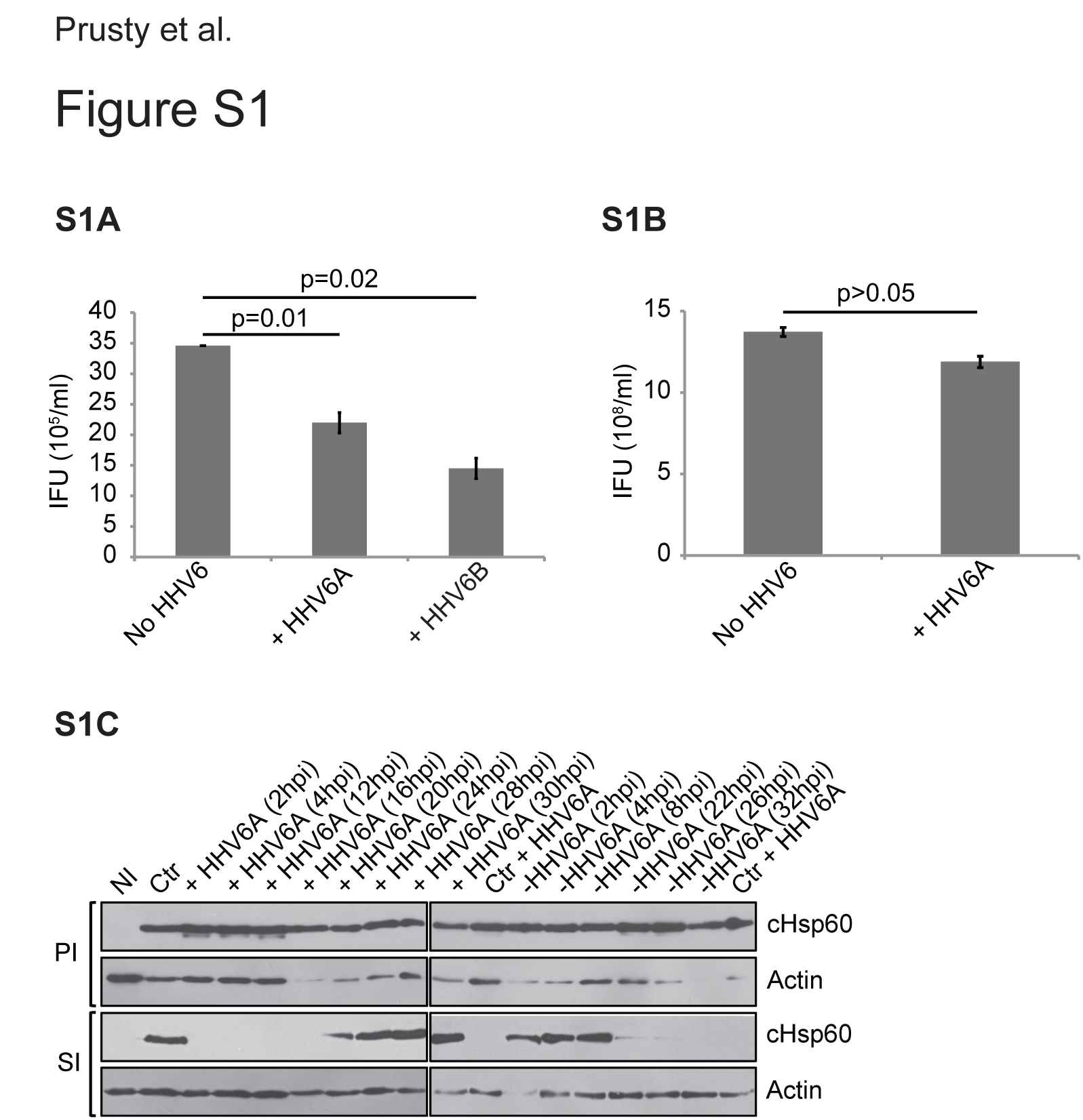

Supplement: Figure S1 — (A,B) HeLa cells were co-infected with HHV6A and 6B and C. pneumoniae (A) or HHV6A and S. negevensis (B) and infectivity was determined after 3 or 4 days post infection, respectively. Cell supernatants were collected 72 h p.i. and were added to freshly growing HeLa cells. At 24 h p.i., cells were fixed and stained for bacterial Hsp60 protein with an antibody against cHsp60 and Cy2-coupled secondary antibody. Inclusion numbers were counted under a fluorescence microscope against the nuclear DAPI staining. IFU, inclusion forming units. Data represent the mean ± SEM of two independent experiments. (C) Early co-infection with HHV6 is necessary for inducing chlamydial persistence. HeLa cells were infected with Chlamydia for 2 h prior to the addition of viral particles for different time points as indicated. In a parallel infection set up, HHV6A was added to Chlamydia-infected cells after 2 h, but subsequently HHV6 was removed from the infection media at the indicated time points. Infectivity assays were performed as described in Material and Methods and immunoblotting was done to check the bacterial Hsp60 protein (cHsp60) expression. PI, primary infection; SI, secondary infection; IFU, inclusion forming units. (TIF) [file pone.0047427.s001.tif]

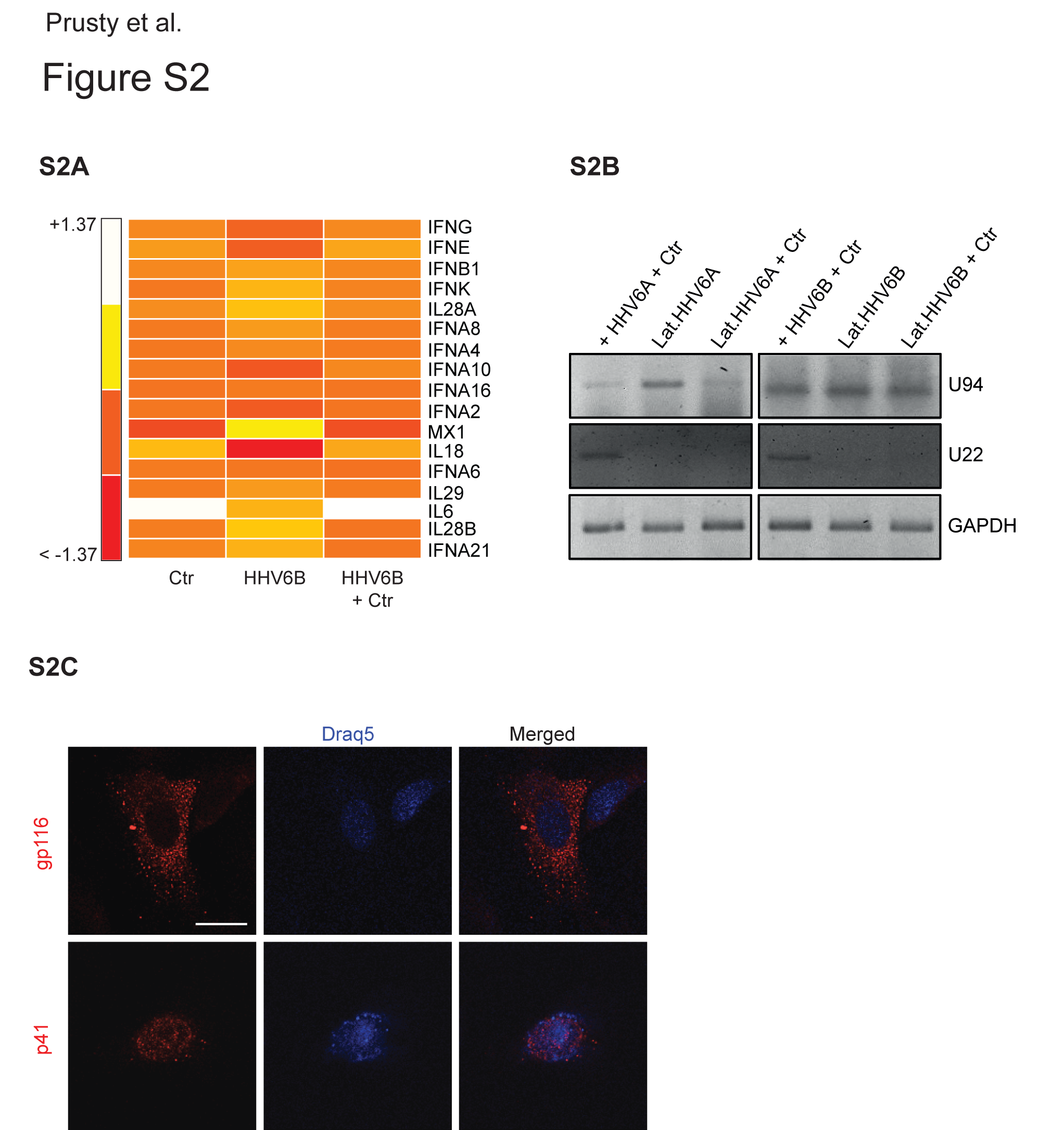

Supplement: Figure S2 — (A) Interferon response is unchanged in single and co-infected cells. Heatmap analysis of microarray data showing differentially expressed interferon genes in HeLa cells under different infection conditions as compared to non-infected cells. Total RNA preparations from non-infected as well as HeLa cells infected with C. trachomatis (Ctr) and/or HHV6B were analyzed. White or red colors indicate differentially up- or down-regulated genes, respectively according to their log2 fold change values. IFNG, Interferon gamma; IFNE, Interferon epsilon; IFNB1, Interferon betta 1; IFNK, Interferon kappa; IL28A, Interleukin 28A; IFNA8, Interferon alpha 8; IFNA4, Interferon alpha 4; IFNA10, Interferon alpha 10; IFNA16, Interferon alpha 16; IFNA2, Interferon alpha 2; MX1, myxovirus resistance 1; IL18, Interleukin 18; IFNA6, Interferon alpha 6; IL29, Interleukin 29; IL6, Interleukin 6; IL28B, Interleukin 28B; IFNA21, Interferon alpha 21. (B) High HHV6 U94 transcription together with the lack of other viral gene transcription demonstrates viral latency. Viral latency was characterized by amplification of HHV6 U94 and U22 transcripts. High transcription of U94 together with absence of U22 transcripts validated that the HHV6 genome was maintained in a latent state. (C) HHV6 glycoproteins are expressed in infected HUVEC cells. HUVEC cells were infected with HHV6A for 72 h and gp116 and p41 were detected by immunostaining using antibodies against the respective proteins and Cy3-coupled secondary antibodies. Draq5 staining was used to stain cellular DNA. Samples were viewed under a confocal laser microscopy. Scale bar, 10 µm. (TIF) [file pone.0047427.s002.tif]

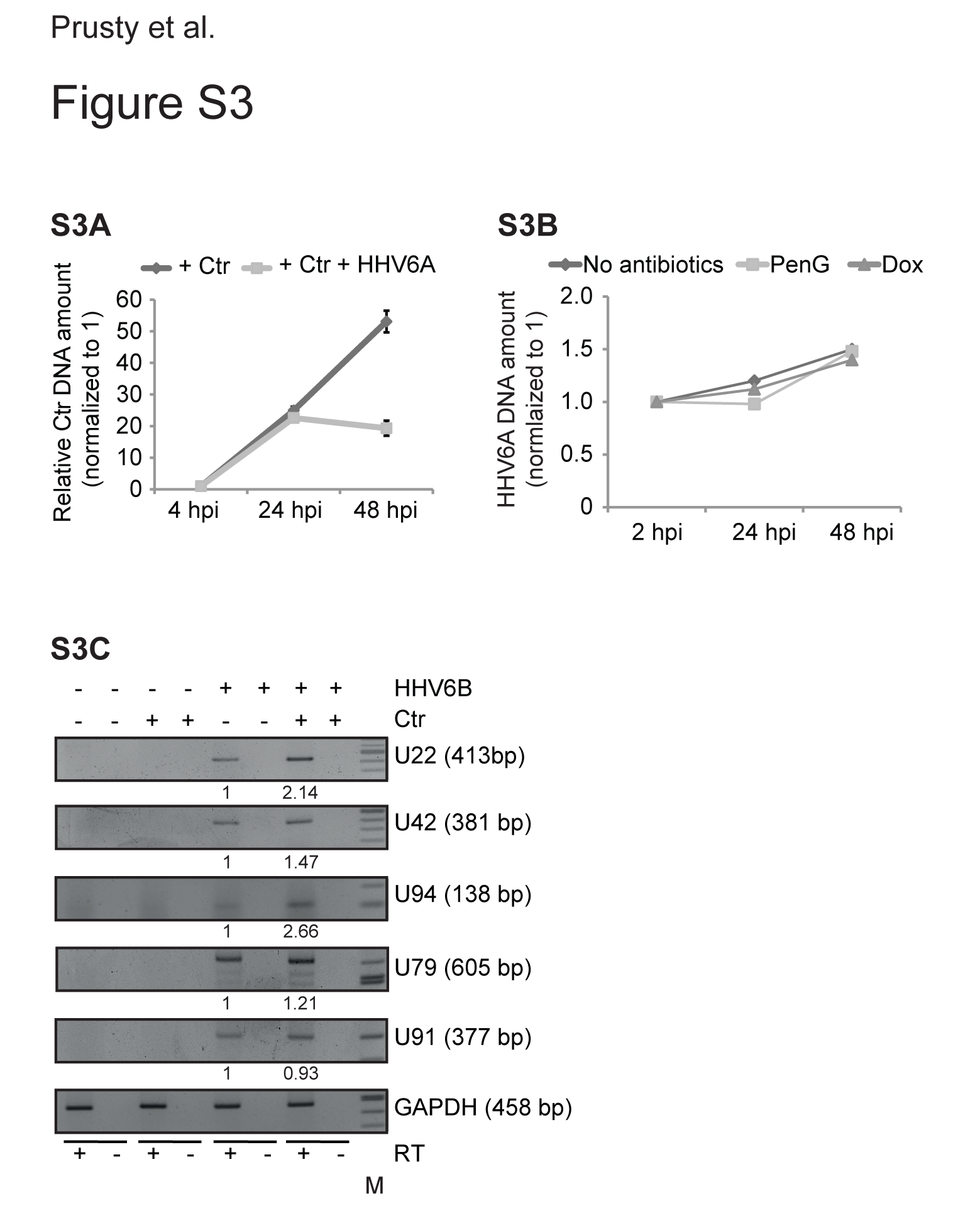

Supplement: Figure S3 — Co-infection of HHV6A and C. trachomatis (Ctr) favors viral survival and entry. (A) Chlamydial replication is down regulated by HHV6. HeLa cells were infected with C. trachomatis and/or HHV6A for different time intervals and DNA was extracted from these cells. Chlamydial DNA was quantified by qPCR, using a primer set against chlamydial LcrH/SycD. Relative viral and Ctr DNA quantity were derived by normalizing the values against 5S rDNA as internal control. In all the graphs, relative values are normalized to 1. Data represent the mean ± SEM of three independent experiments. (B) Penicillin G and Doxycyclin have no effect on single infection with HHV6A. HSB2 cells were infected with HHV6A in absence of any antibiotics. In parallel 2 other sets of HSB2 cells were infected with HHV6A either in presence of 10 U/ml of Penicillin G or 100 ng/ml of Doxycyclin for different times. DNA was extracted and used for qPCR with primers against viral U94 ORF. Relative quantity of U94 level was derived by normalizing against 5s rDNA as internal control. Relative viral DNA values are normalized to 1. Data represent the mean ± SEM of three independent experiments. hpi, hours post infection; dpi, days post infection. (C) HHV6B gene transcription is induced during co-infection with Ctr. HeLa cells were either infected with HHV6A or Ctr alone or co-infected together for 24 h. Total RNA was extracted, reverse transcribed and used for semi-quantitative RT-PCR using primers against viral U22, U42, U79, U91 and U94 ORFs. Amplified products were run on a 2% agarose gel. GAPDH amplification was used as an internal control. Fold change values were derived by dividing respective band intensity with GAPDH band intensity and are mentioned below respective bands. RT, reverse transcriptase; M, marker. (TIF) [file pone.0047427.s003.tif]

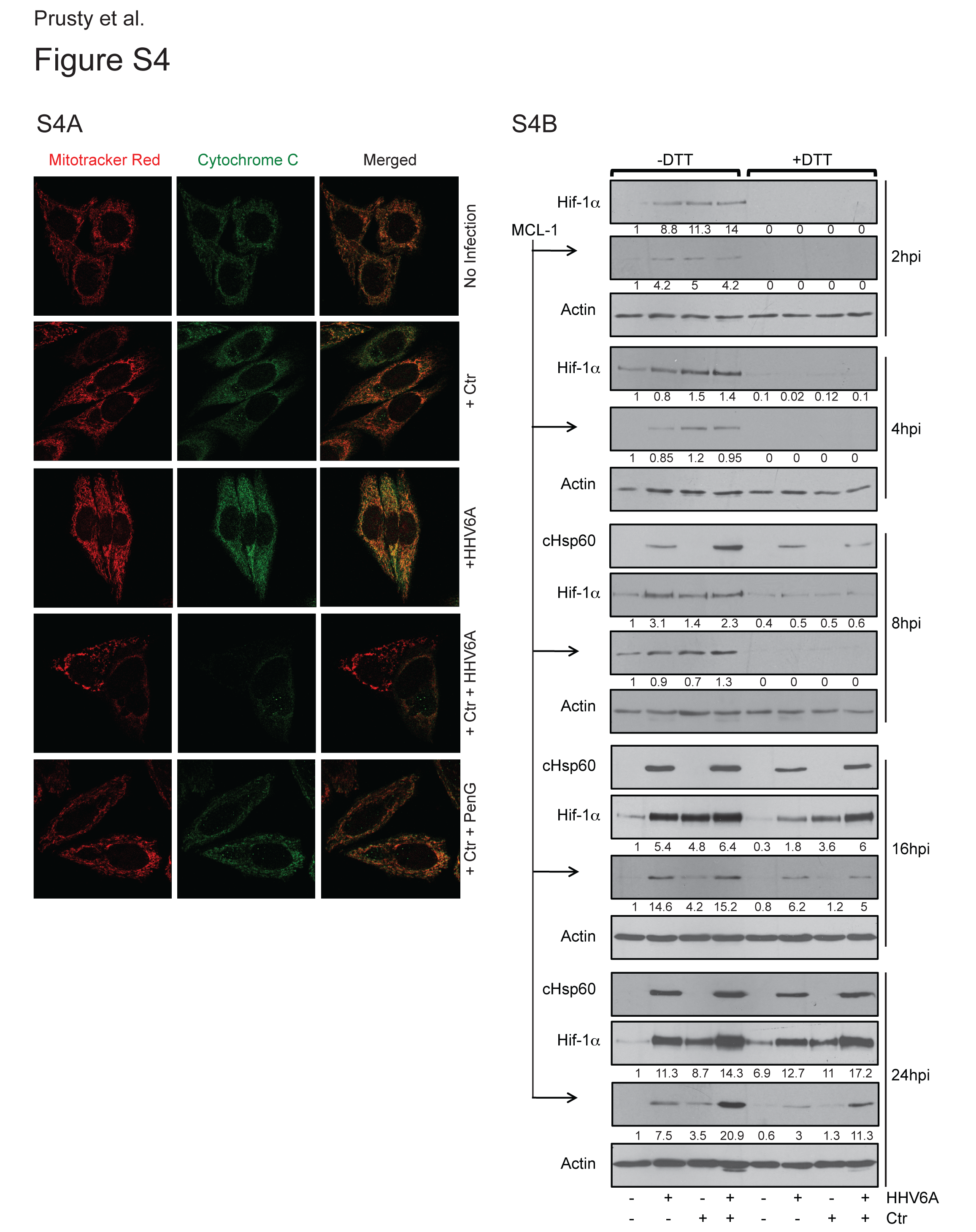

Supplement: Figure S4 — (A) Co-infection of HHV6 and Ctr down regulates host cell mitochondrial membrane potential and induces cytochrome c release. HeLa cells were infected with Ctr and/or HHV6A. As a control, persistence was induced with penicillin G (PenG). Mitochondrial membrane potential was measured in HeLa cells by staining with Mito Tracker (red). Co-staining was done for cytochrome c (green) with using antibody against human cytochrome c and Cy2-coupled secondary antibody (green). Co-localization of mitochondria and cytochrome c was studied under a confocal microscope. (B) HHV-6A and -6B induces Hif-1alpha. HeLa cells were infected with Ctr and/or HHV-6A for different time intervals either in the presence (+DTT) or absence (-DTT) of 1 mM DTT. Chlamydia (cHSP60), Hif-1α and Mcl-1 expression was detected by immunoblotting. Actin was used as a loading control. Fold change values of Hif-1alpha and Mcl-1 was derived by dividing respective values with Actin and are mentioned below each lane. (TIF) [file pone.0047427.s004.tif]

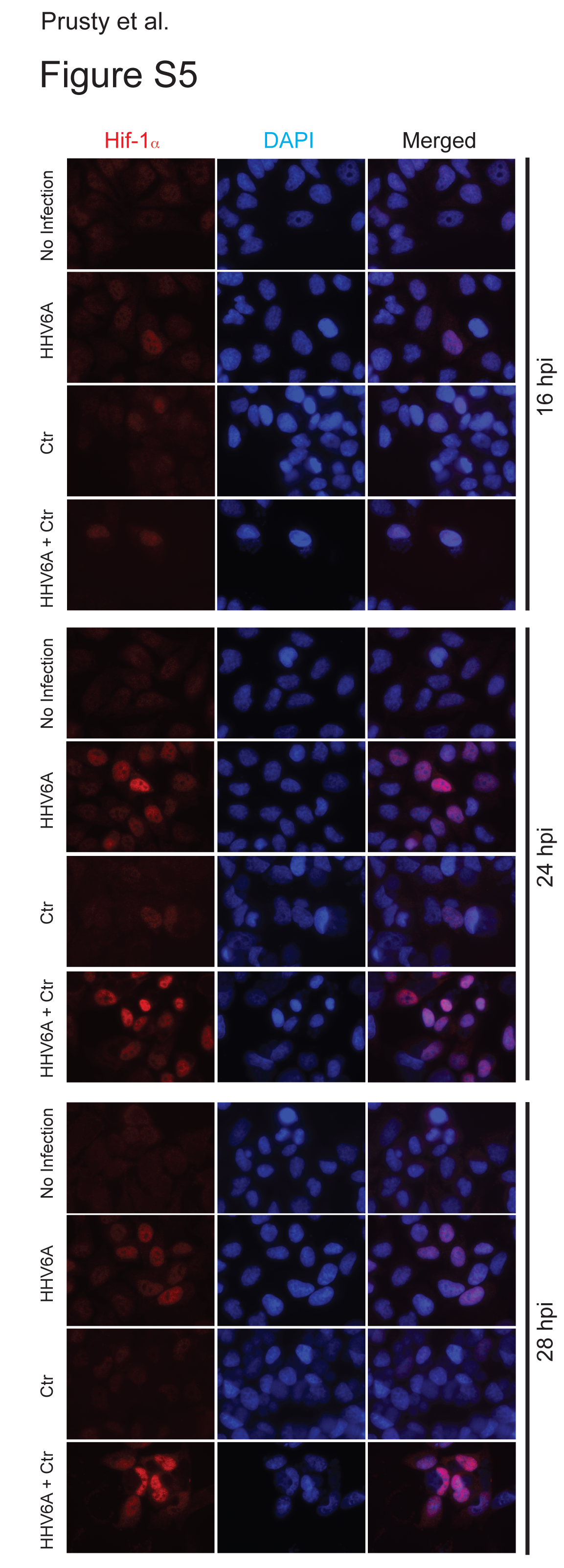

Supplement: Figure S5 — Hif-1α expression is induced during co-infection with HHV6. HeLa cells were infected with Ctr and/or HHV6A for different times. Cells were fixed and stained for Hif-1alpha using an antibody against human Hif-1alpha and Cy3-coupled secondary antibody (Red). Host cell DNA was stained with DAPI (blue). Fluorescence microscopy was used to visualize the localization of Hif-1α. (TIF) [file pone.0047427.s005.tif]

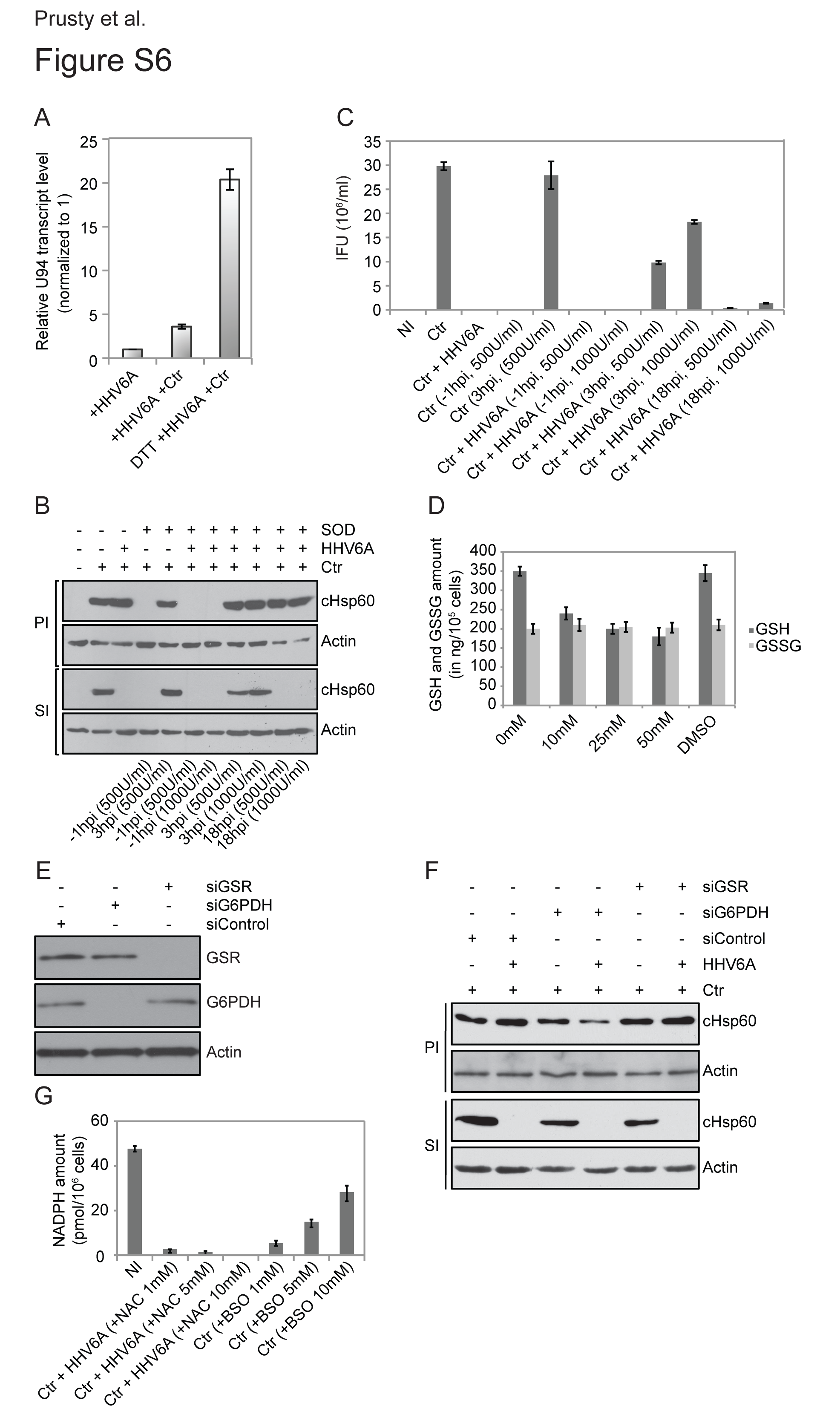

Supplement: Figure S6 — (A) DTT has no negative effect on HHV6 survival inside the host cell during co-infection. HeLa cells were infected either with Ctr or together with HHV6A. Infected cells were supplemented with DTT (1 mM). Total RNA was extracted after 24 h of infection and viral U94 transcript level was quantitated using primers against HHV6A U94. Relative U94 transcript level was derived by normalizing the values against 5s rRNA internal control. Relative U94 values are normalized to 1. Data represent the mean ± SEM of three independent experiments. (B & C) SOD prevents HHV6-mediated chlamydial persistence. HeLa cells were either first treated with SOD (−2 h p.i.) or directly infected either with Chlamydia (Ctr) or together with HHV6A. SOD at different concentrations was added to cells at 3 different time points of infection. Infectivity assay was performed as described in material and methods to check chlamydial infectivity. Immunoblotting (B) was done and inclusion numbers were counted (C) to check chlamydial infectivity. IFU, inclusion forming units. Immunoblot (B) represents one of the three biological replicates. IFU/ml data (C) represent the mean ± SEM of three independent experiments. (D) Total cellular GSH and GSSG level was measured in HeLa cells after 24 hrs of 2-AAPA treatment. Data represent the mean ± SEM of three independent experiments. (E) siRNA mediated gene silencing of G6PDH, GSR was verified by immunoblotting. Data represents one of the three biological replicates. (F) siRNA mediated gene silencing of G6PDH, GSR decreases Chlamydial infectivity. HeLa cells were transfected with 5 nM of G6PDH, GSR siRNAs for 48 hrs. In parallel, a control siRNA pool was also transfected. siRNA transfected cells were then infected with Ctr or together with HHV6A. Infectivity assay and immunoblotting was carried out to check Chlamydial infectivity. siRNA-mediated gene silencing efficiency was checked by using antibodies against G6PDH and GSR. Actin was detected as a loading control [file pone.0047427.s006.tif]
