# Supplementary material for: Imbalanced Oxidative Stress Causes Chlamydial Persistence during Non-Productive Human Herpes Virus Co-Infection
Source: PLoS One. 2012 Oct 15;7(10):e47427. doi: 10.1371/journal.pone.0047427 (PMC3471814; doi:10.1371/journal.pone.0047427)
Supplement: Table S1 — Primers used for semi-quantitative RT-PCR and qRT-PCR. (DOC) [file pone.0047427.s007.doc]

| Gene | Sequence (5’ →3’) | Amplimer size |
| --- | --- | --- |
| **Primers for semi-quantitative RTPCR** | | |
| HHV6.U22 | GGATCCAAAGCAAACCAGCAAGA (forward)  TGGCGGATGGCTAGTGTGCC (reverse) | 413bp |
| HHV6.U42 | AGTTAGTTTCACAGGTGTCAGC (forward)  ACCGAAATCTTTCTTTTACTTGTC (reverse) | 381bp |
| HHV6.U79 | AATGGGTTCTCTAACGGTGGAT (forward)  ATTCATCATGTTGTTGATCTTCGTG (reverse) | 605bp |
| HHV6.U91 | CGTTAAAGATACTGGCATGTCT (forward)  TAAAGTCTCTACTGAAGAAGCA (reverse) | 377bp |
| HHV6A.U94 | ACGGGGACGTGCTAATCCAT (forward)  TCCGGGTGGACCGATAAAAC (reverse) |  |
| GAPDH | TGGATATTGTTGCCATCAATGACC (forward)  GATGGCATGGACTGTGGTCATG (reverse) | 460bp |
| **Primers for qRT** | | |
| HHV6A.U94 | GCGCTCCCGGTGAGTGCATA (forward)  AGGCCCCATGGAGTGGGAGG (reverse) | 110bp |
| HHV6B.U94 | GCGCTCCCGGTGAGTGCATA (forward)  CCCGTGGAGTGGGAGGCAGA (reverse) | 106bp |
| Ctr LcrH/SycD | AGGCGTACGATTTGATCGCCAA (forward)  GCCTACATCTGCTACACCCTGC (reverse) | 136bp |
| 5S rRNA | GTCTACGGCCATACCACCC (forward)  AAAGCCTACAGCACCCGGT (reverse) | 121bp |
| NOX1 | TGCAGCCGCACACTGAGAAAGCA (forward)  GCCATCTGTGGCCTGTCGGCT (reverse) | 150bp |
