# Supplementary material for: Imbalanced Oxidative Stress Causes Chlamydial Persistence during Non-Productive Human Herpes Virus Co-Infection
Source: PLoS One. 2012 Oct 15;7(10):e47427. doi: 10.1371/journal.pone.0047427 (PMC3471814; doi:10.1371/journal.pone.0047427)
Supplement: Materials and Methods S1. — (DOCX) [file pone.0047427.s008.docx]

**Supplemental information**

**Supplementary materials and methods**

**Generation of HeLa cells with latent HHV6 infection**

We created several batches of HeLa cells with latently infected HHV6A and HHV6B. For this, HeLa cells were infected with HHV6A or HHV6B (10^7^ TCID_50_/ml for 10^6^ cells) three times within 5-6 passages. Cells were allowed to grow for 12-15 weeks with regular media change and passaging. After a long cell growth period, latently infected cell lines were characterized by the presence of viral DNA (50-100 copies of viral DNA per 1000 cells, data not shown) without production of viral particles. HHV6 latency was confirmed by the detection of high transcription rates of the latency specific viral gene, U94 in the absence of transcription of other viral genes (Fig. S2B) as previously described [[1](#_ENREF_1),[2](#_ENREF_2)]

**HHV6 virus stock preparation**

Purified virus stocks were prepared from HSB2 and Molt3 suspension cells by the method described by [[3](#_ENREF_3)]. HHV6 infected cells were mixed with uninfected HSB-2 or Molt3 cells at a ratio of 1:10. When more than 80% of cells showed cytopathic effects visible under light microscope, cell-free culture fluid was harvested and filtered through a 0.45-µm filter and the virus was pelleted down by centrifugation at 25,000 x g for 3 h at 4°C. The virus pellet was suspended in cold IMDM media without any antibiotics and frozen at –80°C until further use. The HHV6 titer expressed as the 50% tissue culture infective dose (TCID_50_) was determined by scoring the number of HSB-2 or Molt3 cells exhibiting cytopathic effects. Viral titer and TCID_50_ value was calculated using Reed-Münch formula [[4](#_ENREF_4),[5](#_ENREF_5)] . The virus stock used had a titer of 10^8^ TCID_50_/ml. For viral infection experiments, epithelial cells were seeded for 24 h and then directly added with HHV6 (10^7^ TCID_50_/ml for 10^6^ cells).

**HSV1 virus preparation**

Vero cells were infected with HSV1 at a MOI of 0.01. After 2-3 days (52-58 h) all cells rounded up and showed cytopathic effect. Cells were scraped out and were sonicated on ice. Cell suspension was then centrifuged at 2060 g for 10 min at 4°C to remove cell debris. Collected cell supernatant was further centrifuged at 12000 rpm for 90 min at 4°C using a SW28 or type 19 rotor in the ultracentrifuge to pellet the virus. Virus pellet was resuspended in DMEM media without serum and any antibiotics.

**DNA and RNA extraction**

Total cellular DNA was extracted using DNAzol (Invitrogen) and following manufacturer’s protocol. Total RNA was extracted using TRIZOL (SIGMA) and following manufacturer’s protocol.

**Semi-quantitative RTPCR**

First-strand cDNA synthesis was performed using 200 U of SuperScript II RNase H- reverse transcriptase (Invitrogen, Carlsbad, CA) primed with 5 pmol random hexamers (GIBCO, Gaithersburg, MD) at 42°C for 1 h. RNA-DNA secondary structures were removed by adding 1 U RNase H (USB Corporation, Ohio, USA) to the first strand cDNA products and incubating it at 37°C for 20 min. One microliter of the reverse-transcription products was subjected to 30-35 cycles of PCR in a total volume of 25 µl. The different primers and their sequences with annealing temperature are given in Supplementary Table S1. Amplified PCR products were run on a 2% Agarose gel. When required, the PCR products were purified from agarose gels and cloned into PCR 2.1 vectors (Invitrogen, Carlsbad, CA).

**Quantitative real time PCR (qPCR)**

For qPCR, PerfeCTa™ qPCR SuperMix (Quanta Biosciences) was used and PCR amplifications were done on a StepOnePlus™ real time PCR platform (Applied Biosciences) using manufacturers protocol and SYBR^®^ Green chemistry. Amplified data were analyzed using StepOne™ Software v2.1. Primer details are provided in supplementary table 1.

**Mitotracker staining**

For MitoTracker staining, live cells were washed thrice with PBS and incubated with MitoTracker red dye for 30 min at 37°C. Cells were washed extensively with PBS and fixed for immunostaining as described in the infectivity assay section.

**Microarray analysis of Human genes**

For host cell gene expression analysis, we used two channel microarrays (Agilent-Whole Human Genome, 43271 genes). Infected samples labeled with Cy5 and reference sample (non-infected HeLa cells) labeled with Cy3 were used for hybridization. Three biological replicates per group and three technical replicates per biological replicate were used. Heatmap for host cell interferon mRNA expressions was generated using the R software.

**Antisera and antibodies**

Mouse monoclonal cHsp60 raised against chlamydial recombinant Hsp60 (1:100) (ALX-804-072-R100**,** Enzo Life Sciences, USA), rabbit polyclonal CD46 raised against amino acids 35-328 of CD46 of human origin (sc-9098), mouse monoclonal α-Enolase raised against recombinant α-Enolase of human origin (sc-100812), mouse monoclonal glutathione reductase raised against amino acids 391-510 of glutathione reducatse of human origin (sc-133159) (Santa Cruz Biotechnology, USA) mouse monoclonal actin antibody against human beta-actin.

**References:**

1. Yoshikawa T, Asano Y, Akimoto S, Ozaki T, Iwasaki T, et al. (2002) Latent infection of human herpesvirus 6 in astrocytoma cell line and alteration of cytokine synthesis. J Med Virol 66: 497-505.

2. Rotola A, Ravaioli T, Gonelli A, Dewhurst S, Cassai E, et al. (1998) U94 of human herpesvirus 6 is expressed in latently infected peripheral blood mononuclear cells and blocks viral gene expression in transformed lymphocytes in culture. Proceedings of the National Academy of Sciences of the United States of America 95: 13911.

3. Lu C, Zeng Y, Huang Z, Huang L, Qian C, et al. (2005) Human herpesvirus 6 activates lytic cycle replication of Kaposi's sarcoma-associated herpesvirus. Am J Pathol 166: 173-183.

4. Reed LJ, Muench, L.H. (1938) A simple method of estimating fifty percent endpoints. American Journal of Hygiene 27: 493-497.

5. Turcanova V, Bundgaard B, Höllsberg P (2009) Human herpesvirus-6B induces expression of the human endogenous retrovirus K18-encoded superantigen. Journal of clinical virology 46: 15-19.
